# Review of *Halalaimus* (Nematoda, Enoplida, Oxystominidae) and description of two new species from the abyssal nodule-bearing Clarion-Clipperton Zone

**DOI:** 10.3897/zookeys.1284.187212

**Published:** 2026-07-03

**Authors:** Tania Nara Bezerra, Nic Smol, Renata M. S. Alves, Ellen Pape

**Affiliations:** 1 Marine Biology Research Group, Ghent University, Campus Sterre S8, Krijgslaan 297, 9000 Ghent, Belgium Marine Biology Research Group, Ghent University Ghent Belgium; 2 Symbiosis Environmental Services, SIA Q 4C LT 56 S/N SALA: 214; PARTE 2 L. CEP: 71.200-045, Brasília-DF, Brazil Symbiosis Environmental Services Brasilia Brazil

**Keywords:** Biogeography, deep sea, identification keys, morphological trait analysis, polymetallic nodules

## Abstract

This review examines the geographical distribution, morphology, and taxonomy of the genus *Halalaimus*. It consolidates existing descriptions of the currently 102 valid species, resolves nomenclatural inconsistencies, and introduces two new species: *Halalaimus marcosi***sp. nov**. and *Halalaimus pierri***sp. nov**. *Halalaimus marcosi***sp. nov**. is characterised by its large body, papilliform inner labial sensilla, outer labial setae shorter than cephalic setae spaced one head diameter apart, and a conic-filiform tail with the posterior two-thirds filiform. *Halalaimus pierri***sp. nov**. is distinguished by its very strongly tapered anterior end, indistinct inner labial sensilla, and outer labial setae longer than the cephalic setae. Long-standing challenges in species-level identification, mainly due to incomplete descriptions and confused nomenclature, are addressed through a detailed reassessment that corrects duplicated names, assigns replacement names to misattributed species, and incorporates previously overlooked taxa. *Halalaimus borealis* Gagarin, 2020 is considered a junior homonym and is renamed *H. gagarini***nom. nov**. This study provides interactive global distribution maps for all *Halalaimus* species, a table summarising species ranges and habitat information, and a table listing 36 morphometric and morphological traits per species. Updated identification keys are presented, including a new key for deep-sea species, offering a practical tool for nematode taxonomists and identifiers worldwide.

## Introduction

The genus *Halalaimus* was established by de Man in 1888, with *H. gracilis* as the type species. The specimens were collected from the former island of Walcheren (now a peninsula since the late 19^th^ century), located in the Dutch province of Zeeland at the mouth of the Scheldt estuary, which flows into the North Sea. In his original description, de Man (1888) noted that the species was not rare along the coast of Walcheren. *Halalaimus* de Man, 1888 species can be found all over the world in marine, brackish and terrestrial habitats and in different types of sediment and substrata, revealing high diversity in the deep sea.

*Halalaimus* is distinguished by its characteristic elongated and slender body, as well as by its unique amphid structure, which features a narrow, longitudinally elongated aperture and fovea. These traits clearly separate it from related taxa and facilitate its identification. Species of this genus are found in a wide range of habitats: terrestrial (*H. algeriensis* Coomans & Jacobs, 1983), freshwater (*H. dolgovi* Alekseev & Linnik, 1994, *H. longipharynx* Gagarin & Nguyen Vu Thanh, 2018, *H. parvulus* Gagarin & Nguyen Vu Thanh, 2018, *H. stammeri* Schneider, 1940), brackish (19 valid species), and marine environments (86 valid species). Its broad distribution may be linked to its bacterial feeding mode. In marine environments, *Halalaimus* is found from coastal zones to the deep sea (> 200 m), where it is among the most frequently encountered nematode genera (e.g., Lambshead and Boucher 2003; Vanreusel et al. 2010). Samples often include several *Halalaimus* species, but with few adult specimens per species, as observed by, amongst others, Mawson (1958), Vitiello (1970), Keppner (1992), and Bussau (1993), making species identification challenging.

**Figure 1.**
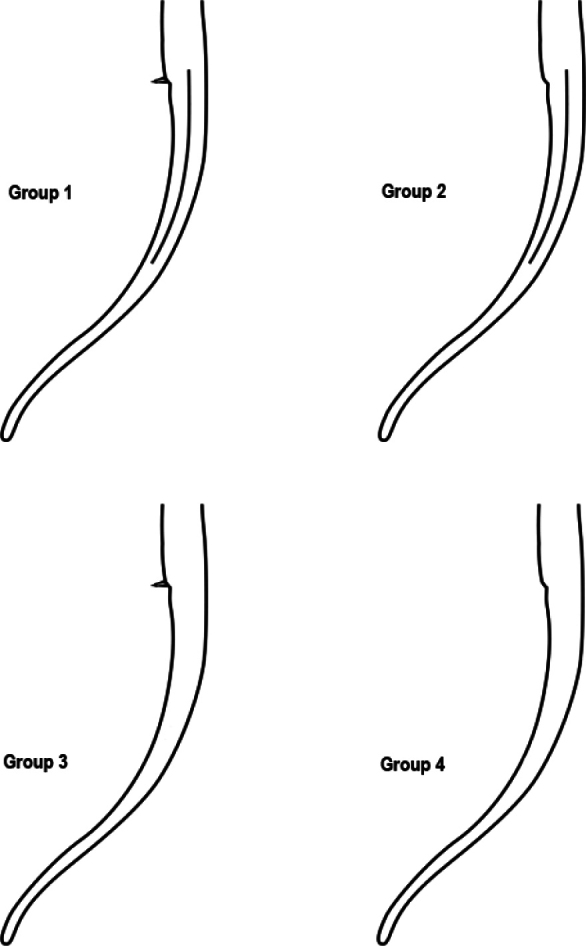
*Halalaimus* groups according to Keppner (1992). Group 1 - Caudal alae and precloacal supplement (setiform or pore) present; Group 2 - Caudal alae present, precloacal supplement (setiform or pore) absent; Group 3 - Caudal alae absent, precloacal supplement (setiform or pore) present; Group 4 - Caudal alae and precloacal supplement (setiform or pore) absent.

Several revisions and identification keys have been developed for *Halalaimus*. Wieser (1953) based his key primarily on amphid width and the distance between the outer labial and cephalic setae. Mawson (1958) observed considerable variation in amphid length and position relative to the anterior end and emphasised the presence of a basal head constriction as a diagnostic character for the subgenus *Tycnodora* Cobb, 1920. Her key to *Halalaimus* s.str. and *Tycnodora* was mainly based on the demanian ratio “a” (above or below 100), the size of outer labial and cephalic setae, and the shape of the tail tip. Wieser and Hopper (1967) highlighted that three species possess a distinct circle of inner labial setae, this feature was later used by Juario (1974) to establish the subgenus *Nualaimus* Juario, 1974. The revision by Keppner (1992) is a landmark work, offering highly detailed descriptions of important morphological traits, supported by clear and informative illustrations, and remains, so far, the best reference for describing new *Halalaimus* species. Based on male morphological traits, Keppner (1992) subdivided *Halalaimus* into four groups according to the presence or absence of caudal alae and precloacal supplements or pores (Fig. 1).

In this paper, we present a morphological and taxonomic revision of the genus *Halalaimus* incorporating currently available data as well as information on the traits not covered by the Keppner (1992) revision. Doing this revision, we created global distribution maps of all *Halalaimus* species based on occurrences in the peer-reviewed scientific literature. We also developed updated and new identification keys. Further, the present manuscript includes the description of two new species from the Clarion-Clipperton Zone, a region of interest for deep-seabed mining for polymetallic nodules.

## Material and methods

### Sampling site

**Figure 2.**
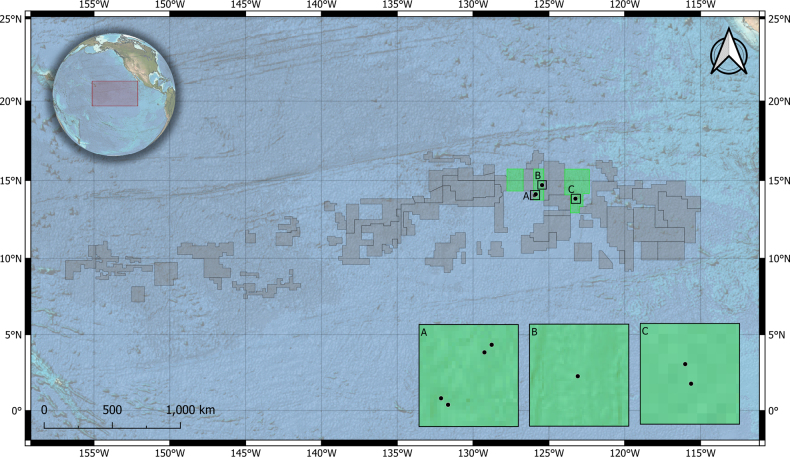
Clarion-Clipperton Zone (CCZ) exploration contract areas for polymetallic nodules (in grey), showing the GSR exploration contract area (divided in three zones) in green, and the sampling stations (black dots) in inserts **A, B** and **C** where the two new *Halalaimus* species were found.

Samples were collected in the Clarion-Clipperton Zone (CCZ) during the GSRNOD15A (September–October 2015) and GSRNOD17 (May–June 2017) expeditions. Three polymetallic nodule (PMN) sites with either nodule-rich or nodule-free sediments were sampled to characterize the environmental baseline as part of a service agreement between Ghent University (UGent) and contractor Global Sea Mineral Resources (GSR). The CCZ is located in the sub-equatorial northeastern Pacific Ocean (Fig. 2). The newly described species were retrieved from the stations listed in Table 1.

**Table 1.** Sampling-station metadata from the GSR exploration contract area where new *Halalaimus* species were discovered. MUC = multicore

**Expedition**	**Site**	**Station**	**MUC**	**Coordinates**	**Water depth (m)**	**Sampling date**	**Species**	**Type specimen**
**GSRNOD15A**	B6S02	NodRich_C	1	13°51'13.85"N, 123°15'18.46"W	4526	23/09/2015	*H. marcosi*	Male holotype
	B4S03	NodRich_A	4	14°7'4.08"N, 125°52'44.76"W	4490	29/09/2015	*H. marcosi*	Female paratype
	B4N01	NodRich_D	1	14°42'22.79"N, 125°27'4.68"W	4504	7/10/2015	*H. marcosi*	Female paratype
**GSRNOD17**	B4S03	NodFree	12	14°03'74.98"N, 125°55'19.01"W	4575	26/05/2017	*H. marcosi*	Male and female paratype
**GSRNOD15A**	B6S02	NodRich_C	3	13°50'44.12"N, 123°15'9.32"W	4522	24/09/2015	*H. pierri*	Male holotype
**GSRNOD17**	B4S03	NodRich_A	19	14°07'34.49"N, 125°52'14.81"W	4500	30/05/2017	*H. pierri*	Female paratype
	B4S03	NodFree	11	14°04'0.088"N, 125°55'44.44"W	4649	26/06/2017	*H. pierri*	Female paratype

### Methods

Undisturbed sediment cores were retrieved using a multicore (MUC) device. Each core (inner diameter: 100 mm) was sliced at 0–5 cm depth and fixed in a formaldehyde–seawater solution at a final concentration of 4%. Meiofauna was extracted by rinsing the sediment over a 32-µm mesh sieve and applying Ludox® (DuPont) HS 40 (density 1.18 g cm^–3^) for three rounds of centrifugation (Burgess 2001). After the final round, the supernatant was preserved in a 4% formaldehyde solution buffered with lithium carbonate and stained with Rose Bengal. Meiofaunal organisms were manually sorted, and 100 nematodes per sample were randomly selected and mounted on slides in glycerine, following De Grisse (1969). When fewer than 100 nematodes were present in a sample, all available individuals were processed.

Identification and descriptions of the two new species were performed using interferential contrast microscopy (Leica DMLS; Leica Microsystems, Wetzlar, Germany). Illustrations were drawn using a camera lucida. Images were captured with a Leica K5C digital camera and processed using Leica LAS X imaging software. Species descriptions followed the guidelines proposed by Braby et al. (2024) and Mokievsky et al. (2024).

Morphometric, habitat, and geographical distributional information was compiled for each described species, and updated identification keys were developed. Morphometric data for the revised species were compiled from the original and subsequent species descriptions.

All measurements were given in micrometres (µm), except for ratios (which are unitless). Curved structures were measured along their arc. Type specimens are deposited in the Zoology Museum of Ghent University – Museum voor Dierkunde (**UGMD**), K.L. Ledeganckstraat 35, 9000 Ghent, Belgium.

All maps presented were built using the General Bathymetric Chart of the Oceans v. 2019 (GEBCO Compilation Group 2019). The International Seabed Authority’s Interactive Map for the contract area outlines are available at: https://www.arcgis.com/home/item.html?id=79137ffc6b284ab29648b07eed37df84.

This work and its nomenclatural acts (newly described species) have been registered in ZooBank, the online registration system of the ICZN.ZooBank - http://zoobank.org/. Life Science Identifiers (LSIDs) are available and can be consulted using any standard web browser.

### Abbreviations used

**L** total body length

**a** total body length / maximum body diameter

**b** total body length / pharyngeal length

**c** total body length / tail length

**c'** tail length / body width at level of anus or cloacal opening

**v** vulva distance from anterior end

**V%** vulva position as % of body length from anterior end

**abd** anal/cloacal body diameter

**cbd** corresponding body diameter

**mbd** maximum body diameter

**HD** head diameter at level of cephalic setae

**ILS** inner labial sensilla

**OLS** outer labial sensilla

**CS** cephalic sensilla

**Amph** from ant. end.: distance from anterior end to anterior edge of amphidial fovea

**B** brackish

**CCZ** Clarion-Clipperton Zone

**DS** deep sea

**GEBCO** General Bathymetric Chart of the Oceans

**GSR** Global Sea Mineral Resources

**GSRNOD15A** name of the 2015 sampling expedition to GSR exploration contract area in the CCZ

**GSRNOD17** name of the 2017 sampling expedition to GSR exploration contract area in the CCZ

**F** freshwater

**M** marine

**N. R** distance from anterior end to anterior limit of nerve ring

**PMN** polymetallic nodules

**Spic L** spicule measured along the median curve

**Spic./abd** spicule length divided by cloacal body diameter

**T** terrestrial

## Results and discussion

### Taxonomic revision of *Halalaimus*

With the discovery of two new *Halalaimus* species in the GSR contract area of the CCZ (Fig. 2), a comprehensive review of the genus was undertaken. Since the erection of the genus *Halalaimus* by de Man (1888), several subgenera were established, based on the following morphological features:

- *Nuada* Southern, 1914: extremely slender body, thick cuticle, rounded head without lips and papillae, 4 submedian setae, head divided from neck by circular groove, indiscernible lateral sense organs (amphids), separating it from *Halalaimus*; no buccal cavity, males without supplements and females with bilobed symmetrical ovaries.
- *Tycnodora* Cobb, 1920: female species with thick, glossy cuticle, 2 circles of outer labial and cephalic sensilla close together, narrow, longitudinally elongated amphids, broad lateral field, and conoid-cylindrical tail.
- *Pachyodora* Wieser, 1953: species with very large amphids, measuring at mid-length approximately 40% of the corresponding body diameter.
- *Halalaimussensu stricto* Wieser, 1953: amphids as in *Tycnodora*: max. 25% of cbd, but with outer labial and cephalic sensilla located far apart.
- *Nualaimus* Juario, 1974: species with inner labial setae instead of papillae.

Schuurmans Stekhoven Jr (1935) synonymised *Nuada* with *Halalaimus* without explanation, as reported by Allgén (1953).

The genus *Halalaimoides* Cobb, 1933 was synonymised with *Halalaimus* by Hope and Murphy (1972).

The subgenus *Tycnodora* was synonymised with *Nuada* by Gerlach and Riemann (1974).

The genus *Acoma* Steiner, 1916, with type species *Acoma borealis* Steiner, 1916, was a junior homonym of *Acoma* Casey, 1890 (Coleoptera), and was replaced by *Nemacoma* by Hope and Murphy (1972). The type species *Nemacoma borealis* (Steiner, 1916) Hope & Murphy, 1972 was treated as a species inquirenda by Gerlach and Riemann (1974).

Lorenzen (1981, 1994) synonymised *Nualaimus*, *Pachyodora* (lapsus: *Pachydora*) and *Nemacoma* Hope & Murphy, 1972 with *Halalaimus*. The synonymisation of *Nemacoma* is based on the original description of the type species *N. borealis* (Steiner, 1916) Hope & Murphy, 1972, with the following characteristics: extremely slender body; tapering at both ends; absence of visible lips or papillae; no buccal cavity; pharynx more than one-third of the body length; ventral gland located slightly anterior to the pharyngeal end; tail tip forming a characteristically spherically swollen shape. Accordingly, the type and only species *Nemacoma borealis* (Steiner, 1916) Hope & Murphy, 1972 becomes *Halalaimus borealis* (Steiner, 1916) Lorenzen, 1981. The status of *Halalaimus*, becoming a genus without subgenera but with many species, is followed by Keppner (1992) and subsequent authors; as did the misspelling of *Pachyodora* (Wieser, 1953) as *Pachydora* (lapsus). We refer to the original spelling, *Pachyodora*.

The species described by Bussau (1993) were, until recently, not included in any key of *Halalaimus*. However his work has recently been regarded as a valid publication (Holovachov, 2020) and therefore we included those *Halalaimus* species in this work.

**Problematic and reinterpreted species**

- Gagarin (2020) described a new species under the name *Halalaimus borealis* Gagarin, 2020, apparently overlooking the valid name *H. borealis* (Steiner, 1916) Lorenzen, 1981 in Lorenzen (1981, 1994). *Halalaimus borealis* Gagarin, 2020 represents a junior homonym; therefore, we propose the new name *Halalaimus gagarini* nom. nov. for this species.
- *Halalaimus ciliocaudatus* Allgén, 1932: the original description of a female *H. ciliocaudatus* Allgén, 1932 from Campbell Island differs markedly in a- and c-values from the male described by Gerlach (1957) from Brazilian mangroves. The most notable differences are the presence of a head constriction, longer setae, and the tail-tip morphology: round and swollen in the original description versus a 14 µm fine terminal tube (probably the spinneret) curved dorsally in Gerlach’s specimen. We therefore consider *H. ciliocaudatus* sensu Gerlach, 1957 a species inquirenda.
- Besides the extensive remarks by Keppner (1992) on *H. gracilis* de Man, 1888, we add the following redescriptions: Allgén (1947) provides only a few measurements without information on the anterior sensilla or somatic and caudal alae; Allgén (1949) refers to 2 juvenile females surrounding another nematode and only provides the length and demanian ratios of one specimen; Allgén (1951) states that cephalic setae could not be located; and in Allgén (1959) only general dimensions are given. In the redescription by Pastor de Ward (1998) the cuticle is striated instead of smooth, and both the inner labial sensilla and the somatic and ornamented caudal alae are absent. We therefore consider all these redescriptions species inquirendae.
- In the original description, *H. isaitshikovi* Filipjev, 1927 is unique in possessing a distinct protrusion of the cuticle around the cloaca; however, none of the later redescriptions refers to this character. Furthermore, *H. isaitshikovi* as described by Allgén (1932) and Schuurmans Stekhoven Jr (1935) lacks lateral alae; the specimen described by Bresslau and Schuurmans Stekhoven (1940) also lacks lateral alae and has a precloacal sensillum (not described by Filipjev 1927). The redescription provided by Allgén (1946) is entirely insufficient to identify the species. In the redescriptions by Platt and Warwick (1983) and Zhang and Huang (2005), ornamented caudal alae are present. Since none of these features were recorded by Filipjev (1927), we consider all these redescriptions species inquirendae.
- In the original description of *H. longicaudatus* Filipjev, 1927, the species is characterised by a small preanal papilla located 2.2 µm anterior to the cloaca and by a 32-µm postcloacal flattening. These characters are not mentioned in the redescriptions by Schuurmans Stekhoven Jr (1946; 1 juvenile), Platt and Warwick (1983) or Zhang and Huang (2005). Moreover, Platt and Warwick (1983) reported similar lengths for the outer labial and cephalic setae and the presence of ornamented caudal alae, whereas Filipjev (1927) described outer labial sensilla shorter (0.8× HD) than the cephalic setae (0.5× HD) and noted the absence of caudal alae. Therefore, we also consider these redescriptions species inquirendae.
- *H. setosus* Timm, 1961: Pastor de Ward (1998) described this species with four cephalic setae instead of six, notwithstanding that six cephalic setae were seen by Timm (1961) and this was confirmed by Rao (1989) and Keppner (1992); therefore, we consider *H. setosus* sensu Pastor de Ward, 1998 as species inquirenda.
- *Halalaimus estuarii* Sinha, Choudhury & Baqri, 1985 was presented at the 2 ^nd^ National Seminar on Marine Intertidal Ecology (Waltair, India), but the species was never formally published and is treated as a nomen nudum.
- In her PhD thesis, Pastor de Ward (1986) described *H. papillifer* and *H. parasetosus*, which were never published in a peer-reviewed journal. These are therefore nomina nuda.
- Blood et al. (1992) listed three species *H. sapeloi*, listed by Wieser (1964) and regarded as nomen nudum by Gerlach and Riemann (1974), *H. cuthemon* and *H. sublatus*, the latter two could not be traced and are therefore also considered nomina nuda.

**Additional taxonomic remarks**

- *Halalaimus capitulatus* Boucher, 1977: in the original description no precloacal sensillum is present, whereas in Platt and Warwick (1983) a sensillum appears in the accompanying drawing, although it is not mentioned in the brief text.
- *Halalaimus leptoderma* Platonova, 1971 was treated as a species inquirenda by Gerlach and Riemann (1974) without justification, but was reinstated as valid by Keppner (1992).
- *H. praestans* Bussau, 1993, the author designated the holotype as male; however, he actually described a female.
- *Halalaimus sagarensis* Sinha, Choudhury & Baqri, 1986 has never been included in any taxonomic revision or identification key. As both a male and female were described, the species is considered valid and included in the key presented in this manuscript.
- *Halalaimus similis* Allgén, 1930 was regarded as a synonym of *H. borealis* Steiner, 1916 by Lorenzen (1981, 1994), based on the bullet-shaped swollen tail tip and other morphological similarities. However, Keppner (1992) treated it as a valid species but did not incorporate *H. borealis* (Steiner, 1916) Lorenzen, 1981 in his list of known species. Comparing the original descriptions and the fact that both species were found in more or less the same geographic region, we follow Lorenzen (1981) and accept *H. similis* as a synonym of *H. borealis*.
- The description of *H. supercirrhatus* Gerlach, 1955 differs from that in Gerlach (1962) in several aspects, including the length of anterior setae, the distance between the two circles of setae, the amphid position, and tail length. Because both descriptions were done by the same author, further evidence is necessary.
- Gerlach and Riemann (1974) listed seven species as species inquirendae, of which two new without giving details about the decision: *H. acuminatus* (Cobb, 1933) Hope & Murphy, 1972 (likely due to its insufficient description of a single male and the lack of illustrations) and *H. leptoderma* Platonova, 1971. Next to these were listed *H. filicaudatus* Allgén, 1959 (insufficiently described according to Gerlach, 1962); *H. filiformis* (Allgén, 1929) by Schuurmans Stekhoven Jr (1935): description based on a juvenile specimen; *H. similis* Allgén, 1930 (considered insufficiently known according to Wieser (1953) and *H. strandi* Allgén, 1934 by Wieser (1953): description based on a juvenile specimen, and *H. southerni* Allgén, 1953 by Mawson (1958). To that list Keppner (1992) added *H. droebachiensis* Allgén, 1931 and we recognise another 16 species inquirendae (Table 3).
- Gerlach and Riemann (1974) listed three species as nomina nuda: *H. annulatus* Wieser, 1960; *H. sapeloi* Wieser, 1964 and *H. simiferus* Wieser, 1960. These species were only mentioned in ecological papers (Table 3).

**Overview of current accepted and unaccepted species**

According to Nemys eds. (2026), there are 101 valid species, five species inquirendae and eight nomina nuda. Our investigation resulted in 102 valid species (Table 2), 23 species inquirendae, and eight nomina nuda (Table 3).

**Table 2.** Accepted *Halalaimus* species: authority, Keppner male key groups (* = interpretation of the authors of this paper), females and habitat.

**Species name**	**Authority**	**Group**	**Habitat**
*H. absconditus*	Bussau, 1993	Fem	M – Deep sea
*H. abyssus*	Bussau, 1993	4*	M – Deep sea
*H. aciculus*	Gagarin & Nguyen Vu Thanh, 2014	1	M
*H. aedificandistudiosus*	Bussau, 1993	4*	M – Deep sea
*H. alatus*	Timm, 1952	2	M
*H. algeriensis*	Coomans & Jacobs, 1983	Fem	B, T
*H. americanus*	Keppner, 1992	1	M
*H. amphidellus*	Vitiello, 1970	Fem	M
*H. amphistrius*	Vitiello, 1970	Fem	M
*H. anne*	Sergeeva, 1972	4*	M, B
*H. arundinaceus*	Bussau, 1993	Fem	M – Deep sea
*H. bayensis*	Keppner, 1992	1	M
*H. borealis*	(Steiner, 1916) Lorenzen, 1981	Fem	M
*H. brachyaulax*	Mawson, 1958	Fem	M
*H. brevispiculum*	Sergeeva, 1973	4	M
*H. brimi*	Keppner, 1992	2	M
*H. bulbocaudatus*	Keppner, 1992	1	M
*H. capitulatus*	Boucher, 1977	4	M
*H. caroliniensis*	Chitwood, 1936	4	M
*H. ciliocaudatus*	Allgén, 1932	4	M
*H. cirrhatus*	Gerlach, 1953	3	M
*H. climactericus*	Wieser, 1953	Fem	M
*H. comatus*	Wieser, 1953	Fem	M
*H. cubanus*	Andrássy, 1973	3	M
*H. curvicaudatus*	Juario, 1974	3	M
*H. delamarei*	Vitiello, 1970	3	M – Deep sea
*H. deseadensis*	Pastor de Ward, 1998	Fem	M
*H. diacros*	Mawson, 1958	4	M
*H. dimorphus*	Turpeenniemi, 1997	1	B
*H. diplocephalus*	Filipjev, 1927	Fem	M
*H. dolgovi*	Alekseev & Linnik, 1994	3	F
*H. durus*	Gagarin & Nguyen Vu Thanh, 2004	2	B
*H. egregius*	Bussau, 1993	Fem	M – Deep sea
*H. filicollis*	Timm, 1961	4	M
*H. filicorpus*	Vitiello, 1970	4	M – Deep sea
*H. filum*	Gerlach, 1962	2	M, B
*H. fletcheri*	Mawson, 1958	4	M
*H. florescens*	Gerlach, 1967	4	M
*H. floridanus*	Keppner, 1992	1	M, B
*H. gagarini* nom. nov.	(Gagarin, 2020) This paper	4	B
*H. gerlachi*	Keppner, 1992	2	M
*H. gidanensis*	Nasira & Turpeenniemi, 2002	2	M
*H. gracilis*	de Man, 1888	2	M, B, Deep sea
*H. horridus*	Gerlach, 1956	3	M
*H. isaitshikovi*	(Filipjev, 1927) Schuurmans Stekhoven, 1935	4	M
*H. jaltensis*	Sergeeva, 1973	4	M, B
*H. leptoderma*	Platonova, 1971	4	M
*H. leptosoma*	(Southern, 1914) Schuurmans Stekhoven, 1935	4	M
*H. lineatoides*	Timm, 1961	2	M
*H. lineatus*	Timm, 1961	2	B
*H. longamphidus*	Huang & Zhang, 2005	2*	M
*H. longicaudatus*	(Filipjev, 1927) Schneider, 1939	4	M
*H. longicollis*	Allgén, 1932	4	M
*H. longinquus*	Bussau, 1993	Fem	M – Deep sea
*H. longipharynx*	Gagarin & Nguyen Vu Thanh, 2018	4	B, F
*H. longisetosus*	Hopper, 1963	Fem	M
*H. longistriatus*	Timm, 1961	4	M
*H. lutarus*	Vitiello, 1970	4	M
*H. luticolus*	Timm, 1961	4	B
*H. macquariensis*	Mawson, 1958	4	M
*H. marcosi* sp. nov.	This article	1	M – Deep sea
*H. marri*	Mawson, 1958	3	M
*H. meyersi*	Wieser & Hopper, 1967	4	M
*H. minimus*	Gagarin, 2016	1	B
*H. minor*	Gagarin & Nguyen Vu Thanh, 2004	2	B
*H. minusculus*	Tchesunov, 1978	3	M
*H. monstrocaudatus*	Vitiello, 1970	3	M
*H. nigrilapidarius*	Boucher, 1977	3	M
*H. oblongus*	Bussau, 1993	4	M – Deep sea
*H. orientalis*	Gagarin, 2016	1	B
*H. pachyderma*	(Filipjev, 1927) Schuurmans Stekhoven, 1935	4	M
*H. pachydermatus*	(Cobb, 1920) Schneider, 1939	Fem	M
*H. pachyodoroides*	Vitiello, 1970	4	M
*H. papillifer*	Gerlach, 1956	4	M
*H. paracomatus*	Keppner, 1992	1	M
*H. parafletcheri*	Keppner, 1992	3	M
*H. parvulus*	Gagarin & Nguyen Vu Thanh, 2018	4	F
*H. parvus*	Chitwood, 1936	4	M
*H. pierri* sp. nov.	This article	2	M – Deep sea
*H. ponticus*	Filipjev, 1922	4	M
*H. praestans*	Bussau, 1993	Fem	M – Deep sea
*H. rectispiculatus*	(Platonova, 1971) Gerlach & Riemann, 1974	4	M
*H. relatus*	Gerlach, 1967	2	M
*H. sagarensis*	Sinha, Choudhury & Baqri, 1986	4	B
*H. sarsi*	Gerlach, 1967	2	M
*H. scleratus*	Timm, 1952	Fem	M
*H. setosus*	Timm, 1961	4	M
*H. shinkai*	Shimada, Takeda, Tsune & Murakami, 2020	2	M – Deep sea
*H. sobakini*	Sergeeva, 1973	1	M
*H. stammeri*	Schneider, 1940	Fem	F
*H. striatus*	Gerlach, 1956	3	M
*H. supercirrhatus*	Gerlach, 1955	4	M
*H. talaurinus*	Leduc, 2023	2	M – Deep sea
*H. tarjani*	Keppner, 1992	1	M
*H. tenuicapitatus*	Filipjev, 1946	4	M
*H. terrestris*	Gerlach, 1959	3	M, B
*H. thalassinus*	Keppner, 1992	1	M
*H. turbidus*	Vitiello, 1970	4	M
*H. variabilis*	Keppner, 1992	1	B
*H. vietnamicus*	Gagarin, 2016	1	B
*H. wodjanizkii*	Sergeeva, 1972	4	M
*H. zenkevitshi*	Filipjev, 1927	4	M

**Table 3.** Previous and new unaccepted *Halalaimus* species with their habitats; new changes indicated by *.

**Species name**	**Status**	**Habitat**
*H. acuminatus* (Cobb, 1933) Hope & Murphy, 1972	sp. inq.	M
*H. ciliocaudatus* sensu Gerlach, 1957	sp. inq.*	B
*H. droebachiensis* (Allgén, 1931)	sp. inq.	M
*H. filicaudatus* (Allgén, 1959) Gerlach, 1962	sp. inq.	M
*H. filiformis* (Allgen, 1929) Schuurmans Stekhoven, 1935	sp. inq.	M
*H. gracilis* sensu Allgén, 1947	sp. inq. *	M
*H. gracilis* sensu Allgén, 1949	sp. inq. *	M
*H. gracilis* sensu Allgén, 1951	sp. inq. *	M
*H. gracilis* sensu Schuurmans Stekhoven, 1935	sp. inq	M
*H. gracilis* sensu Allgén, 1959	sp. inq. *	M
*H. gracilis* sensu Pastor de Ward, 1998	sp. inq. *	M
*H. isaitshikovi* sensu Allgén, 1932	sp. inq. *	M
*H. isaitshikovi* sensu Schuurmans Stekhoven, 1935	sp. inq. *	M
*H. isaitshikovi* sensu Bresslau & Schuurmans Stekhoven, 1940	sp. inq. *	M
*H. isaitshikovi* sensu Allgén, 1946	sp. inq. *	M
*H. isaitshikovi* sensu Platt & Warwick, 1983	sp. inq. *	M
*H. isaitshikovi* sensu Zhang & Huang, 2005	sp. inq. *	M
*H. longicaudatus* sensu Schuurmans Stekhoven, 1946	sp. inq. *	M
*H. longicaudatus* sensu Platt & Warwick, 1983	sp. inq. *	M
*H. longicaudatus* sensu Zhang & Huang, 2005	sp. inq. *	M
*H. setosus* sensu Pastor de Ward, 1998	sp. inq. *	M
*H. similis* (Allgén, 1930) Wieser, 1953	sp. inq.	B
*H. southerni* (Allgén, 1953) Mawson, 1958	sp. inq.	M
*H. strandi* (Allgén, 1934) Wieser, 1953	sp. inq.	M
*H. annulatus* – Wieser, 1960	nom. nud.	M
*H. cuthemon* – Blood et al., 1992	nom. nud. *	M
*H. estuarii* – Sinha, Choudhury & Baqri, 1985	nom. nud. *	B
*H. papillifer* – Pastor de Ward, 1986	nom. nud. *	M
*H. parasetosus* – Pastor de Ward, 1986	nom. nud. *	M
*H. sapeloi* – Wieser, 1964; Blood et al., 1992	nom. nud.	M
*H. simiferus* – Wieser, 1960	nom. nud. *	M
*H. sublatus* – Blood et al., 1992	nom. nud. *	M

### Morphological notes on the genus *Halalaimus*

#### Cuticle

The cuticle is smooth or presents fine to coarse transverse striations, especially in the tail region, or can be clearly sclerotised as in *H. scleratus*. It is possible that all species have a striated cuticle, but in some cases the striations are beyond the resolving power of light microscopes, as noted by Platt and Warwick (1983). Occasionally, punctations are observed (e.g., *H. floridanus* and *H. pierri* sp. nov.) and in one species (*H. longistriatus*), the cuticle bears 36 longitudinal lines. In the head region, the cuticle is typically very thin and never striated but usually thickens substantially from the level of the cephalic sensilla posteriorly, sometimes forming a constriction (*H. comatus*; *H. diacros*; *H. marcosi* sp. nov.). Mid-body thickness can reach up to 40–52% of cbd in *H. talaurinus*.

In some species, modifications of the lateral field into lateral alae or somatic alae occur, starting anteriorly from the amphidial region and extending toward the tail; in other species, they are present only in the caudal region and referred to as caudal alae. The width of the lateral alae varies from 0.10 to 0.33 of mbd. In *H. alatus*, the caudal alae extend from the cloacal region to halfway down the tail and terminates in a surface groove or pit surrounded by heavy sclerotisation. Caudal alae are often ornamented in males; ornamentation in females is only described for *H. paracomatus*.

Internal cuticular modifications (vermiculations) appear in the body and tail regions of some males, located ventrally (e.g., *H. americanus*) or dorsally relative to the lateral lines.

#### Sensory structures

Metanemes. Within Oxystominidae, only orthometanemes are known (Lorenzen 1981, 1994). The presence or absence of metanemes is rarely reported in species descriptions, with exceptions found in Bussau (1993) and Leduc (2023). Bussau (1993) observed no metanemes, whereas Leduc (2023) detected orthometanemes with indistinct caudal and frontal filaments in *H. talaurinus*.

Anterior sensilla. The typical pattern of the anterior sensilla consists of an inner circle of six inner labial sensilla (ILS), which are papillae or setae but often not discernible, followed by six outer labial sensilla (OLS), usually setae, and a third circle of four cephalic sensilla (CS), mostly setae. The latter have been historically referred to as subcephalic, nuchal, or cervical setae. In *H. leptoderma* and *H. pachyderma*, no anterior sensilla were observed (possibly broken?), and *H. leptosoma* presents only four cephalic setae. *Halalaimus filicollis* and *H. setosus* each have six cephalic setae, as confirmed by Rao (1989). We consider these two additional setae as subcephalic setae that have moved into the cephalic circle. The development of different juvenile stages could demonstrate this transition, as shown in Linhomoeidae (Hendelberg, 1979) and Xyalidae (Lorenzen, 1978). In *H. monstrocaudatus*, OLS and CS are shorter in females; in *H. supercirrhatus*, OLS and CS are equal in females, but OLS > CS in males.

Genuine cervical setae are found in *H. supercirrhatus*. Cervical, somatic, and caudal sensilla (setae and papillae) are present in *H. talaurinus*. Somatic setae are usually absent but present in *H. sobakini* and *H. caroliniensis*. *Halalaimus delamarei* has rows of short papilliform setae along the lateral fields. Somatic cuticular pores or pits may also occur, arranged in longitudinal lines (*H. talaurinus*, *H. variabilis*).

Amphids. Amphid structure and terminology are described in detail by Riemann et al. (1970). The amphidial slit (*fovea amphidialis*) is a flat, groove-shaped depression filled to varying degrees with a gelatinous body (*corpus gelatum amphidialis*), as seen in *H. sarsi*. At the bottom of the fovea lies the porus *canalis amphidialis*, connected to the subcuticular *fusus amphidialis* and extending posteriorly via the *nervus amphidialis*. The external opening is called the *apertura amphidialis*. These structures are well illustrated in *H. algeriensis*. In *H. deseadensis*, two or three pores are visible inside the fovea. The fovea’s length, width, and anterior position vary. In some species (e.g., *H. cirrhatus*, *H. climactericus*, *H. tarjani*), the anterior border of the fovea reaches the level of the cephalic setae, whereas in others it is more posterior. Width can range from very narrow (*H. supercirrhatus*) to broad (0.15–0.40 cbd), as in *H. delamarei* and *H. ponticus*.

#### Digestive system

The pharynx is cylindrical and slightly widens posteriorly (*H. aedificandistudiosus*) or may become pear-shaped (*H. abyssus*, *H. egregius*, *H. praestans*), stamp-shaped (*H. absconditus*), or triangular (*H. pachyodoroides*), but never ends in a muscular bulb. The pharyngeal wall is sometimes described as crenate (e.g., *H. algeriensis*), and three glands are often visible with nuclei near the pharyngeal base. In *H. talaurinus* gland ducts extend to the amphidial aperture, though openings are indistinct. Pharyngeal glands are also visible in *H. pachyderma*.

The cardia may be flat (*H. longicaudatus*) or triangular (*H. talaurinus*), partially encased anteriorly in pharyngeal tissue and surrounded posteriorly by intestinal tissue.

A long rectum or prerectum is often reported (e.g. *H. longicaudatus*, *H. stammeri*), with rectal glands observed in *H. longicaudatus*.

#### Secretory-excretory system

The secretory-excretory system is seldom mentioned in the literature. When described, the renette cell is anterior to the pharynx base (*H. dimorphus*, *H. longistriatus*, *H. longicaudatus* or posterior to it (*H. caroliniensis*). The excretory pore is often near the amphidial fovea (*H. meyersi*, *H. pachyodoroides*), or behind it, before the nerve ring (*H. ponticus*) or near mid-pharynx (*H. parvus*).

#### Reproductive system

Details of the reproductive system especially of the males, with the exception of the spicules and gubernaculum, were often overlooked.

Males are diorchic, with opposed outstretched testes, reflexed in *H. marcosi* sp. nov., positioned right or left of the intestine. Only one testis is reported in *H. sagarensis* and *H. alatus*. The system is well described in *H. dimorphus*, *H. aciculus*, and *H. pachyderma*. Precloacal papillae or sensilla may occur, and a postcloacal flattening is noted in *H. longicaudatus*. In *H. sobakini*, a precloacal papilla is linked to a subcuticular gland. Testes may differ in shape (*H. vietnamicus* has a straight anterior and bent posterior testis). Spicules are usually equal, with variable shape, curvature, and velum; length ranges from 1 to 2 abd. In *H. parafletcheri*, spicules differ in width. The gubernaculum ranges from rudimentary (*H. leptoderma*) to gutter-shaped (*H. nigrilapidarius*) or complex (*H. florescens*), with a keel-like and lateral extensions (*H. americanus*, *H. bayensis*, *H. tarjani*), or cuff shaped with sleeves around the spicules (*H. papillifer*). Some species bear a dorsal apophysis (*H. cubanus*, *H. gerlachi*) and *H. pierri* sp. nov. a caudal apophysis. Sperm cells are described in *H. talaurinus* and *H. dimorphus*, the latter showing large sperm in the anterior testis and smaller in the posterior. Turpeenniemi (1998) studied sperm ultrastructure in *H. dimorphus*.

The female reproductive system is didelphic, amphidelphic, with antidromously reflexed ovaries, usually on opposite sides of the intestine. In *H. longicollis* Allgén, 1932, only the posterior gonad is developed, with the ovary dorsally reflexed. *Halalaimus minusculus* and *H. longamphidus* are described with outstretched ovaries. Developmental stages are well illustrated in *H. algeriensis*, showing a transition from outstretched to reflexed ovaries, raising the question of maturity in specimens with outstretched ovaries. A spermatheca is mentioned for *H. dolgovi* and *H. talaurinus*; a pre-uterus (= spermatheca?) is noted in *H. ponticus*. Egg dimorphism is documented in *H. dimorphus*. *Halalaimus marcosi* sp. nov. presents a structure comparable to a spermatheca.

#### Tail

The tail is conical-cylindrical or conical-filiform, the ratio of the conical part versus cylindrical/filiform part varies and it can have coarse transverse striations in the cylindrical part. The tip can be pointed, blunt, swollen, or bifid (with two terminal appendages). Caudal glands are confined within the tail; a spinneret is present or not determined.

#### Sexual dimorphism

Sexual dimorphism is observed in the length of anterior sensilla (*H. monstrocaudatus*, *H. supercirrhatus*), amphid size (*H. turbidus*, *H. nigrilapidarius*), and tail tip shape (*H. zenkevitshi*).

### Morphometric table

The online morphometric traits table https://doi.org/10.5281/zenodo.19406582 contains details of all described *Halalaimus* species available at the time of writing based on the scientific literature. Comparison of the morphometrics and diagnostic characters of the different species and their subsequent redescriptions reveal frequent discrepancies between the original descriptions and those provided by later authors.

The following morphometric parameters are listed for all described species: total body length; a, b, c and c' ratios; V%; presence or absence of a head capsule and head constriction; length of the ILS, OLS and CS; distance between the OLS and CS; head diameter; amphideal fovea; distance from the anterior edge of the amphid to the anterior end; spicules; gubernaculum; abd; ratios of setae length/HD, OLS–CS distance/HD, foveal length/HD and fovea distance/HD; spicules length/abd; shape of the gubernaculum; presence or absence of precloacal supplements; cuticle striations, pores or setae; somatic and caudal alae; ornamentation; vermiculations; structure of the reproductive system; tail and tail tip.

Most *Halalaimus* species (87%) have a body length between 1–3 mm; 12 species are shorter than 1 mm, the smallest being *H. minor* (504–569 µm). Seven species exceed 3 mm, with *H. leptosoma* being the longest (5600–5660 µm). The demanian ratios vary considerably: a = 26–226; b = 2–9; c = 2–22; and c' = 4–106. Species with an a-value > 200 include *H. filicorpus* (213) and *H. leptosoma* (226). Most species have a very long pharynx, reflected in b-values below 5, whereas *H. filicorpus* has the shortest pharynx. Many species have very long tails, including *H. filum*, *H. meyersi* and *H. egregius*. *Halalaimus monstrocaudatus* clearly has the proportionally longest tail (c = 2.2; c' = 106), while *H. supercirrhatus* has the shortest relative tail (c = 21.5; c’ = 7). A few species can be distinguished by their bifurcate tail tip: *H. aedificandistudiosus*, *H. brimi*, *H. diacros*, *H. filicollis*, *H. fletcheri*, *H. cfr fletcheri*, *H. longamphidus*, and *H. parafletcheri*.

The cuticle is very thick in *H. cubanus* (2.5 µm), *H. isaitshikovi* (3 µm), *H. pachydermatus* (1/6 cbd) and *H. pachyderma* (48% cbd). Additional details such as dots, pores or setae are found in 13 (mostly recently described) species. Bussau (1993) paid particular attention to cuticular pores, which occur in many of the deep-sea species from the Peru Basin; the deep-sea species *H. talaurinus* has eight rows of pores. In *H. setosus*, two pores are located in front of the amphid and three pores are present inside the amphideal fovea. Setae occur in *H. gidanensis*, *H. marcosi* sp. nov., *H. sobakini*, *H. shinkai* (arranged in rows) and *H. thalassinus* (cervical, somatic, and caudal setae). Only two species possess longitudinal striations: *H. gidanensis* and *H. longistriatus* (up to 36 longitudinal lines).

Lateral somatic alae are observed in 21 species, and in *H. minor* these are cross-striated. Caudal alae occur in 29 species and are ornamented in 18 of them. In *H. alatus*, the caudal alae terminate in a surface groove/pit surrounded by heavy sclerotisation. Vermiculations are observed only in *H. americanus*, *H. bayensis*, *H. floridanus*, *H. thalassinus* and *H. variabilis*, and are also present in the females of *H. floridanus*.

A head capsule and/or head constriction is reported for only a few species; however, a capsule can often be recognised in drawings but not mentioned in the description. In most *Halalaimus* species the inner labial sensilla are invisible under light microscopy; when visible, they are very small and papilliform in 15 species, and setiform in nine species (excluding cfr. *fletcheri*), including *H. gracilis*. Unfortunately, this feature has been overlooked in many later redescriptions of the type species, such as those by Platt and Warwick (1983) and Pastor de Ward (1998), which makes these redescriptions questionable.

In most species (50 species), the outer labial sensilla and cephalic sensilla are equal in length; the outer labial sensilla are shorter in 31 species and longer in 21 species. The position of these two sensillar circles is usually close (< 1 head diameter) or very close in 57 species, is approximately one head diameter apart in 22 species, and is widely separated (> 1 head diameter) in 19 species. *Halalaimus setosus* has six cephalic setae. Some species are easily recognised by their very long setae: *H. horridus* (OLS = 3 HD), *H. florescens* (CS = 4 HD), *H. floridanus* (CS = 4 HD), *H. meyersi* (> 4 HD), *H. longisetosus* (CS = 5 HD) and *H. supercirrhatus* (5–8 HD).

The amphideal fovea varies greatly in length (17–172 µm). In most species it ranges between 20–50 µm; eight species have amphids < 20 µm, while 27 species have amphids > 50 µm. The longest fovea (172 µm) occurs in *H. egregius*. The anterior end of the fovea may lie close to the head or near the base of the cephalic setae as in *H. cirrhatus*, *H. climactericus* and *H. pachydermatus*, or far posterior, as in *H. capitulatus* (10× HD), *H. americanus* (12× HD) and *H. aciculus* (14× HD).

The shortest spicules occur in *H. minor* (6 µm) and the longest in *H. shinkai* (98 µm). Only in *H. minor* are the spicules shorter than the anal body diameter; in *H. alatus* the spicules length equals the anal body diameter. In most species the spicules length is 1–2 abd; it exceeds 2 abd in *H. cirrhatus*, *H. filicorpus*, *H. longipharynx*, *H. nigrilapidarius* and *H. parvulus* (respectively 2–3 abd; 2.3 abd; 2.2–2.4 abd; 1.9–2.3 abd, and 2–2.3 abd).

A gubernacular apophysis is rare in the genus *Halalaimus* and is present only in *H. alatus*, *H. cubanus*, *H. florescens*, *H. gerlachi*, *H. longicollis*, *H. minor*, *H. minusculus*, *H. pierri* sp. nov., and *H. sarsi*.

Only ~ 30% of *Halalaimus* species possess precloacal supplements, typically a single sensillum (seta). However, *H. dolgovi* and *H. minusculus* have two sensilla (2 setae). In *H. cubanus* and *H. longicaudatus*, a precloacal pore is observed; *H. variabilis* has a precloacal pore, and *H. sobakini* has a precloacal gland and duct. Only *H. minor* has three postcloacal caudal papillae.

The morphometric table demonstrates that taxonomic studies of Halalaimus published so far have placed considerably more emphasis on measurements than on the presence or absence of key morphological characters. It is also clear that many authors rely primarily on recent publications rather than consulting the original descriptions. This further indicates that, although identification keys are practical and easy to use, their application must be approached with caution and verified carefully.

#### Additional remarks

Turpeenniemi (1997) was the first to include microphotographs in species descriptions, documenting 12 males and 12 females from an unusually large sample, giving a good insight of the variability of the species. Since then, subsequent authors (Pastor de Ward 1998; Gagarin and Nguyen Vu Thanh, 2014; Gagarin 2016; Shimada et al. 2020; Leduc 2023) have followed this approach and microphotographs are nowadays standard.

Molecular data for the genus *Halalaimus* are available, but the existing DNA sequences (mainly 18S) represent only a small fraction of total species diversity. This limitation can be attributed to the low number of specimens typically collected per species. In the public databases of BOLD Systems (Barcode of Life Data System) and GenBank, in addition to sequences assigned only to the genus level, sequences are currently available for *H. gracilis*, *H. lineatoides*, and *H. luticolus*.

### Ecological notes on the genus *Halalaimus*

**Figure 3.**
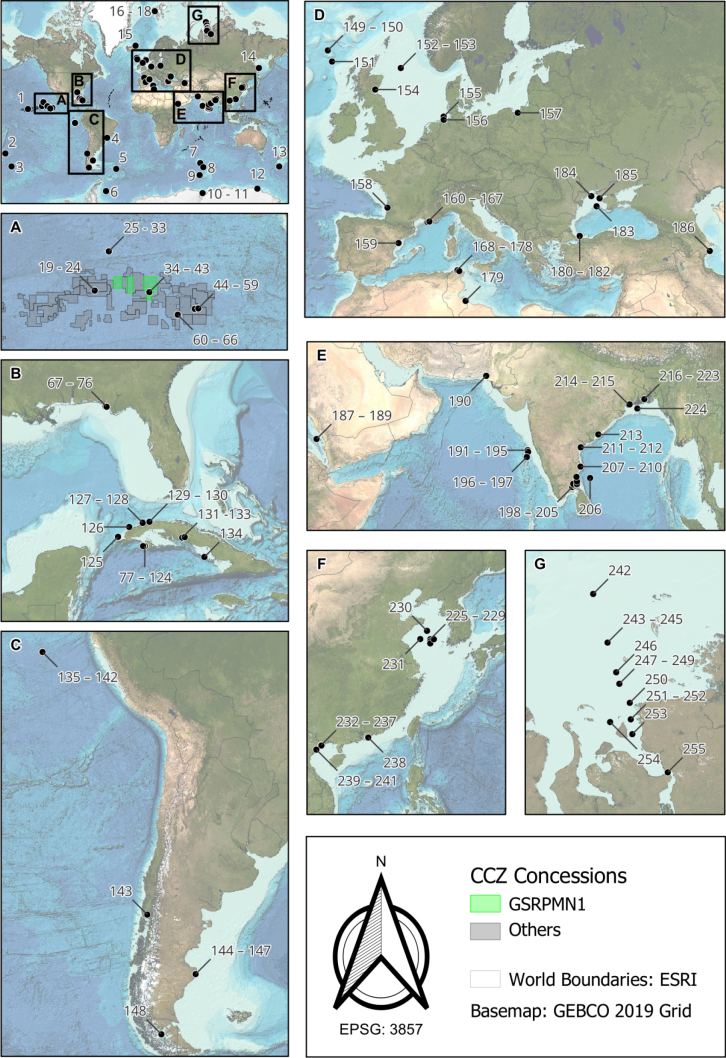
Type localities of valid species of *Halalaimus*, where all species numbers correspond to those listed in in the supplementary material found in the depository Zenodo: https://doi.org/10.5281/zenodo.18600678. An interactive version of this map is available at https://alvesrms.web.app/NematodeMap .

Our comprehensive revision highlights the wide geographical distribution and ecological versatility of the genus *Halalaimus* (Fig. 3). It is a cosmopolitan genus, recorded from marine, brackish, freshwater, and even terrestrial environments. Species of *Halalaimus* inhabit both muddy and sandy sediments, are found from shallow waters to the deep sea and occur in different habitats such as coral, algae, seagrass bed, etc. The distribution table (https://doi.org/10.5281/zenodo.18600678) summarises the known geographical and habitat distribution of all described species. Based on currently available scientific literature all original descriptions, subsequent redescriptions and distributions mentioned in published ecological studies all collected data are displayed in this table. An interactive distribution map of all the *Halalaimus* species can be found at: https://alvesrms.web.app/NematodeMap. Among the 15 species identified from deep-sea environments (see key deep-sea species), 12 appear to be restricted to the deep sea and have not been reported from other habitat types (*H. absconditus*, *H. abyssus*, *H. aedificandistudiosus*, *H. arundinaceus*, *H. egregius*, *H. longinquus*, *H. marcosi* sp. nov., *H. oblongus*, *H. pierri* sp. nov., *H. praestans*, *H. shinkai*, *H. talaurinus*). Among these species some build microtubes from mineral and detritus particles (*H. absconditus*, *H. aedificandistudiosus*, *H. arundinaceus*). These structures may be underreported due to sampling and/or extraction methods. The status of *H. gracilis* as deep-sea species (Allgén 1959; Baishnab et al. 2023) is exceptional. *Halalaimus gracilis*, shows the broadest known distribution, being reported from more than 20 countries across different continents and occurring across a range of water depths (from littoral to abyssal), sediment types (sandy, silty, and muddy), and habitats. However, we hypothesise that these records may include misidentifications, since many are based on ecological surveys, and even in some taxonomic studies the setiform nature of the inner labial sensilla, a key diagnostic character, is not mentioned (e.g., Allgén 1947, 1959; Pastor de Ward 1986, 1998; Zhang and Huang 2005). Nonetheless, due to the lack of consistent morphological information in the available literature, this hypothesis cannot be confirmed.

Of the 12 deep sea-exclusive species, 11 were exclusively found in association with polymetallic nodule (PMN) areas. *Halalaimus absconditus*, *H. abyssus*, *H. aedificandistudiosus*, *H. arundinaceus*, *H. egregius*, *H. longinquus*, *H. oblongus*, and *H. praestans* were, to date, only found in the Peru Basin and the CCZ (Bussau 1993; Pape et al. 2017). *Halalaimus shinkai* Shimada et al., 2020; *H. marcosi* sp. nov., and *H. pierri* sp. nov. are, to date, restricted to the CCZ. Only one deep sea exclusive species, *H. delamarei* Vitiello, 1970, has also been reported from a non-nodule area: the Cassidaigne Canyon in the Mediterranean Sea by Vitiello (1970). Although the genus *Halalaimus* is reported from the Central Indian Ocean Basin, also a nodule-bearing region, the specimens were, unfortunately, not identified to species (Singh et al. 2014). Because most previous qualitative studies have focused on deep-sea nematodes at the genus level, it remains unclear whether their species-level composition is similar worldwide (Bussau 1993).

### Systematics

**Class: Enoplea Inglis, 1983**

**Subclass: Enoplia Pearse, 1942**

**Order: Ironida Hodda, 2007**

**Suborder: Oxystominina Siddiqi, 1983**

**Superfamily: Oxystominoidea Chitwood, 1935**

**Family: Oxystominidae Filipjev, 1918**

**Subfamily: Halalaiminae De Coninck, 1965**

#### Genus Halalaimus

Taxon classificationAnimaliaEnoplidaOxystominidae

de Man, 1888

BF26E06D-8E2D-58E3-B0FD-00C4DF818C7B

##### Note.

*Halalaimus* (generic diagnosis emended after Keppner 1992) Enoplida, Oxystominidae.

##### Type species.

*Halalaimus gracilis* de Man, 1888.

##### Diagnosis.

Body long and slender, distinctly attenuated at both extremities. Cuticle ranging from thin to very thick; thin in the head region, sometimes forming a “cap” and clearly thickening posterior from the level of the cephalic sensilla, sometimes forming a visible constriction. Cuticle smooth or bearing fine to coarse transverse striations; occasionally with longitudinal lines. Cuticular somatic setae, pits, or pores may be present (usually arranged in longitudinal lines). Lateral field often forming lateral alae. Males often with caudal alae, which may be ornamented and occasionally display vermiculations ventrally or dorsally to the lateral line. Anterior sensilla arranged in three separate circles: six inner labial sensilla (papillae or setae), often indistinct; six outer labial sensilla (typically setae); and four cephalic sensilla (typically setae). Amphid with a markedly elongated longitudinal fovea. Pharynx cylindrical, widening posteriorly. Excretory-secretory system with renette cell located at the level of pharyngeal widening, and pore situated near the amphidial fovea. Male reproductive system diorchic (rarely monorchic) with opposed testes. Spicules straight or slightly curved; gubernaculum usually present. Precloacal sensillum or papilla present or absent. Female reproductive system didelphic, amphidelphic with reflexed ovaries. Tail conical-cylindrical or conical-filiform, with the cylindrical portion either smooth or transversely striated. Tail tip pointed, blunt, swollen, or bifid. Caudal glands located within the tail. Spinneret present or undetermined. Sexual dimorphism may occur in the length of the anterior sensilla, the amphid, and the tail.

From the samples collected in the CCZ (see Fig. 2, Table 1), two new species of the genus *Halalaimus* are described: *H. marcosi* sp. nov. and *H. pierri* sp. nov. All deep sea *Halalaimus* species, including the two newly described ones, can be accurately identified using the identification key for the genus *Halalaimus* provided in this paper.

#### Halalaimus marcosi sp. nov.

Taxon classificationAnimaliaEnoplidaOxystominidae

D58C0C73-1799-5B0D-A8AC-A9501D98974C

https://zoobank.org/15C2FB84-12C0-48E3-8408-465D183D8171

**Figure 4.**
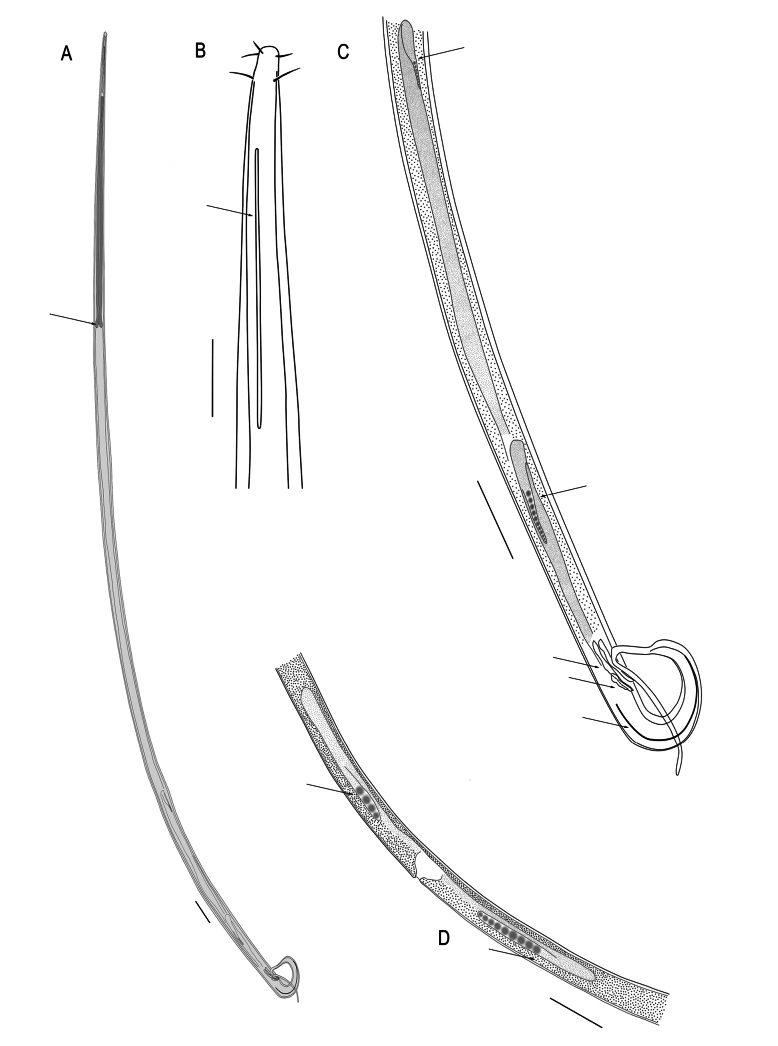
*Halalaimus marcosi* sp. nov. Holotype male. **A**. Habitus, arrows pointing to pear shaped end of pharynx; **B**. Anterior end, arrows pointing to the amphid; **C**. Posterior end, arrows pointing to anterior and posterior reflexed testes, spicules, gubernaculum, and caudal alae. Paratype female; **D**. Reproductive system, arrows pointing to the anterior and posterior reflexed ovaries. Scale bars: 50 µm (**A, C, D**), 100 µm (**B**).

**Figure 5.**
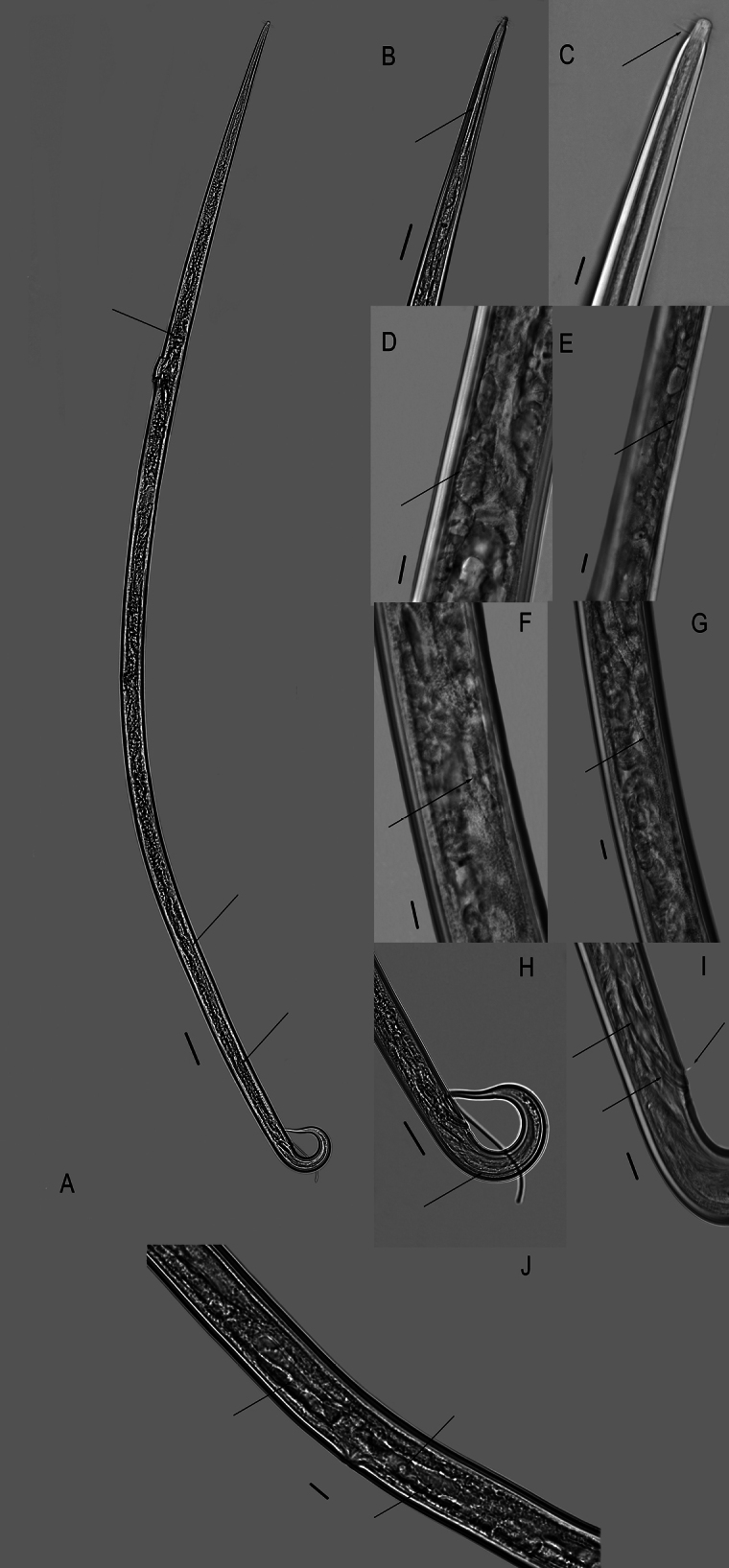
*Halalaimus marcosi* sp. nov. Holotype male. **A**. Habitus, arrows pointing to the pharyngeal bulb; anterior and posterior testes; **B**. Anterior end, arrow points to the amphid; **C**. Anterior end arrows points to the cephalic setae; **D**. End of pharynx, arrow pointing to the posterior pear-shaped widening; **E**. Arrow pointing to the lateral differentiation; **F**. Anterior testis, arrow pointing to the reflexed end; **G**. Posterior testis, arrow pointing to the reflexed end; **H**. Posterior end, arrow pointing to the caudal alae; **I**. Arrows pointing to the spicules, gubernaculum and pre-cloacal sensilla. Paratype female; **J**. Female reproductive system. Arrows pointing to the end of the reflexed ovaries and spermatheca. Scale bars: 50 µm (**A**); 25 µm (**B, H**); 7.5 µm (**C, D, E, F, G, I**); 10 µm (**J**).

Figs 4, 5, Tables 1, 4

##### Type material.

***Holotype*** male (Inventory UGMD_104484). ***Paratypes*** 1 male (Inventory UGMD_1004485); 3 females (Inventory UGMD_1004486, UGMD_1004487 and UGMD_1004488), are deposited in the voucher collection of the Ghent University Museum Zoology Collections - UGent.

**Table 4.** Morphometrics of *H. marcosi* sp. nov. and *H. pierri* sp. nov. All measurements are in µm, except ratios which have no units.

	***Halalaimus marcosi* sp. nov**.					***Halalaimus pierri* sp. nov**.		
	**Holotype ♂**	**Paratype ♂**	**Paratypes ♀ (n = 3)**			**Holotype ♂**	**Paratypes ♀ (n = 2)**	
**L**	**1699**	**1742**	**1970**	**1848**	**1955**	**1622**	**1492**	**1520**
Head diameter at cephalic setae	5	6	4	4	5	4	4	5
Body diameter at anterior end of amphid	9	10	7	7	8	6	5	6
Length of outer labial setae	4	4	3	3	4	2	3	3
Length of cephalic setae	6	6	4	4	5	1	2	2
Length amphideal fovea	72	73	71	75	74	85	85	85
Body diameter- at the middle of amphid	13	12	13	13	14	10	9	10
Distance of amphid from anterior end	26	23	22	23	25	41	33	37
Amphid width	1.8	2	2	2	2	1.5	1	1
Nerve ring from anterior end	281	285	268	273	278	84 ?	137	130
Nerve ring cbd	21	22	21	22	22	13	12	13
Pharynx length	458	460	450	440	450	509	392	420
Pharyngeal width at the base	17	17.5	17	16	17	12	11	12
Pharynx cbd at base	26	28	24	24	25	20	18	20
Maximal body diameter (mbd)	29	31	33	31	32	22	20	21
Anal/Cloacal diameter (abd)	21	23	21	21	22	14	11	12
Spicules length	45	44				21		
Spicules/abd	2.1	2.2				1.5		
Gubernaculum lenght	21	20				12		
Length pre-cloacal sensillum	3	3				5		
Tail length	247	248	252	250	252	212	213	212
a	58.5	56.1	59.6	63	61	74	75	76
b	3.7	3.8	4.3	4.4	4.3	3.2	3.8	3.6
c	6.9	7	7.8	7.8	7.8	7.7	7	7
c'	11.7	10.7	12	12	11.5	15	19.3	18
v			1024	979	1015		746	768
V%			52	50	52		50	51
Vulva cbd			28	27	28		21	21

##### Type locality and habitat.

CCZ, Pacific Ocean. Deep sea sediment dominated by silt, containing polymetallic nodules. *Halalaimus marcosi* sp. nov. was found in samples from two different expeditions to the GSR contract area in the CCZ, i.e. GSRNOD15A and GSRNOD17, in four different stations: NodFree, NodRich_A, NodRich_C and NodRich_D. Water depth around 4500 m, from 0–5 cm (Table 1) of sediment dominated by silt and clay particles.

##### Description.

Male holotype. Body slender, tapering at both extremities. Total length 1699 µm. Cuticle thick, ≤ 4 µm at cloacal region; generally smooth, with striations in tail region. Lateral differentiation present along body, starting at the end of the amphid and extending to beginning of posterior third of tail. Caudal lateral fields not ornamented. Anterior end distinctly set off, with very thin cuticle and basal head constriction. Cuticle thickening from level of cephalic setae to mid-third of tail. Cuticle thickness: 2.5 µm at mid-amphid, 4 µm anterior to cloaca (~ 20% of cloacal body diameter), 0.9 µm at mid-third of tail. Cuticle pores not observed. Metanemes not observed. Labial region rounded. Six inner labial papillae. Six outer labial setae, 4 µm long. Four cephalic setae, 6 µm long. OLS and CS separated by 5 µm. Amphideal fovea elongated, 72 µm long and ~ 2 µm wide. Short somatic setae (~ 1 µm) present near amphid, at mid-body, and near posterior end of amphid. Pharynx muscular, cylindrical, with posterior pear-shaped widening. Cardia embedded in intestine. Nerve ring at 57% of pharynx length. Secretory-excretory system not observed. Reproductive system diorchic, with opposed and reflexed testes. Posterior testis right of intestine, anterior testis left of intestine. Spicules paired, symmetrical, slightly curved, 45 µm long. Gubernaculum 21 µm long, slightly curved, partially enclosing spicules laterally. Precloacal sensillum 2.5 µm long, located anterior to cloaca. Tail 243 µm long, conical-filiform; posterior half filiform. Tail tip blunt. Three caudal glands present, confined in the tail region. Spinneret not observed.

Females. Similar to males but longer. Reproductive system didelphic, amphidelphic, reflexed ovaries. Spermatheca proximally at each ovary branch. Vulva situated slightly after mid-body. Vagina perpendicular, slightly cuticularised. No vaginal glands observed. Three caudal glands present, not visible in all specimens. Spinneret not observed.

Juveniles. Similar to adults in most morphological features. The total length of the examined juveniles (*n* = 3) ranged from 1369–1457 µm. The limits of the amphids were not clearly discernible. The measurements of pharynx = 441 µm, mbd = 24–28 µm, abd = 21 µm, and tail length = 192–196 µm correspond well with the proportions observed in adults. Caudal alae were already visible in juvenile specimens.

##### Diagnosis.

*Halalaimus marcosi* sp. nov. is characterised by a slender body tapering towards both extremities, with total length ranging from 1699–1970 µm. The cuticle is generally smooth along the body, with fine annulations at the tail tip, although not visible in all specimens. Somatic alae are clearly visible. The head is distinctly set off and has a thinner cuticle anterior to the cephalic setae, forming a basal head constriction. The amphid is located approximately five head diameters from the anterior end. Inner labial sensilla are papilliform; outer labial and cephalic sensilla are setiform. Cephalic setae are spaced one head diameter apart. The pharynx is elongated and muscular, with a posterior pear-shaped widening. The cardia is embedded in the intestine. The male reproductive system is diorchic, with opposed and reflexed testes. Spicules are slightly curved; the gubernaculum is also slightly curved. A single precloacal sensillum is located immediately anterior to the cloacal opening. No cuticular pore is observed. The female reproductive system is didelphic, amphidelphic, with reflexed ovaries. Three caudal glands are present, located within the tail but not visible in all specimens. The tail is elongated, with a filiform tip, and shows annulations in two specimens. Based on the presence of caudal alae and a precloacal supplement, *H. marcosi* sp. nov. belongs to Group 1 in the classification proposed by Keppner (1992).

##### Differential diagnosis.

*Halalaimus marcosi* sp. nov. can be distinguished from all previously described species of the genus by a unique combination of morphological characters. These include its large body size (1699–1970 µm), the presence of six inner labial papillae, outer labial setae shorter than cephalic setae, both circles of setae spaced approximately one head diameter apart, a thick cuticle (≤ 4 µm), a pharynx with a posterior pear-shaped widening, and somatic and caudal lateral differentiation that is not ornamented. A notable feature of *H. marcosi* sp. nov. is the presence of reflexed testes, and the presence of a precloacal sensillum. The tail is conical-filiform with a blunt tip. Among the 13 species currently placed in Group 1 of Keppner’s (1992) classification (characterised by the presence of caudal alae and precloacal supplements), nine species have a body length shorter than 1500 µm (*H. minimus*, *H. vietnamicus*, *H. dimorphus*, *H. tarjani*, *H. orientalis*, *H. bulbocaudatus*, *H. bayensis*, *H. floridanus*, and *H. paracomatus*). Three species (*H. comatus*, *H. thalassinus*, and *H. sobakini*) exceed 2000 µm in length. Only *H. variabilis*, along with the largest individuals of *H. floridanus*, approaches the body length of *H. marcosi* sp. nov. (ca. 1700 µm); however, both species possess very long cephalic setae (4–5 HD), in contrast to the shorter setae of *H. marcosi* sp. nov. Based on the diagnostic characters used in the updated key for Group 1—particularly the length, aspect, and position of sensilla and amphids—*H. marcosi* sp. nov. appears most similar to *H. tarjani*. However, *H. marcosi* sp. nov. differs by having papilliform inner labial sensilla, whereas *H. tarjani* presents them as short setae. Additionally, *H. marcosi* sp. nov. lacks ornamentation on the caudal alae, which are distinctly ornamented in *H. tarjani*. Among the deep sea species (deep sea key) it can be recognisable by having and papilliform inner labial sensilla, somatic lateral and caudal alae present, gubernaculum without apophysis, precloacal sensillum present and a non-bifurcate tail tip,

##### Etymology.

The new species is named after the younger and only brother of the first author, Marcos Aurélio Campinas Bezerra.

#### Halalaimus pierri sp. nov.

Taxon classificationAnimaliaEnoplidaOxystominidae

8041887F-2532-5E09-890D-BBCAA496518A

https://zoobank.org/598F1807-47AD-4A97-871B-96C5D0841C1F

**Figure 6.**
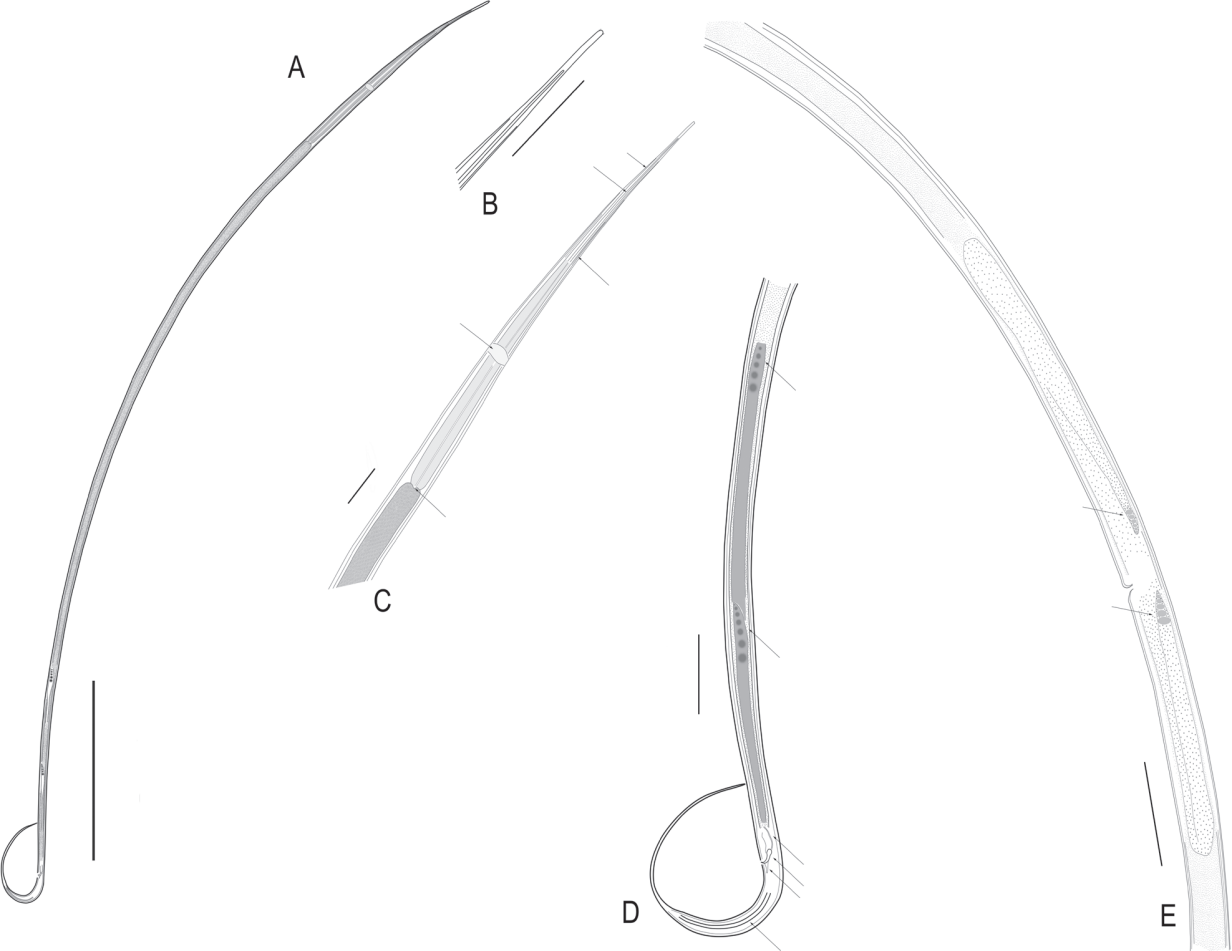
*Halalaimus pierri* sp. nov. Holotype male. **A**. Habitus; **B**. Zoom of anterior end; **C**. Anterior end, arrows pointing to beginning of cuticle enlargement, amphid, lateral differentiation, nerve ring and cardia; **D**. Posterior end, arrows showing the end of the outstretched anterior and posterior testes, spicules, dorsal apophyses and caudal alae. Paratype female; **E**. Reproductive system, arrows showing the end of the reflexed anterior and posterior ovaries. Scale bar: 200 µm (**A**); 50 µm (**B, C, D**); 25 µm (**E**).

**Figure 7.**
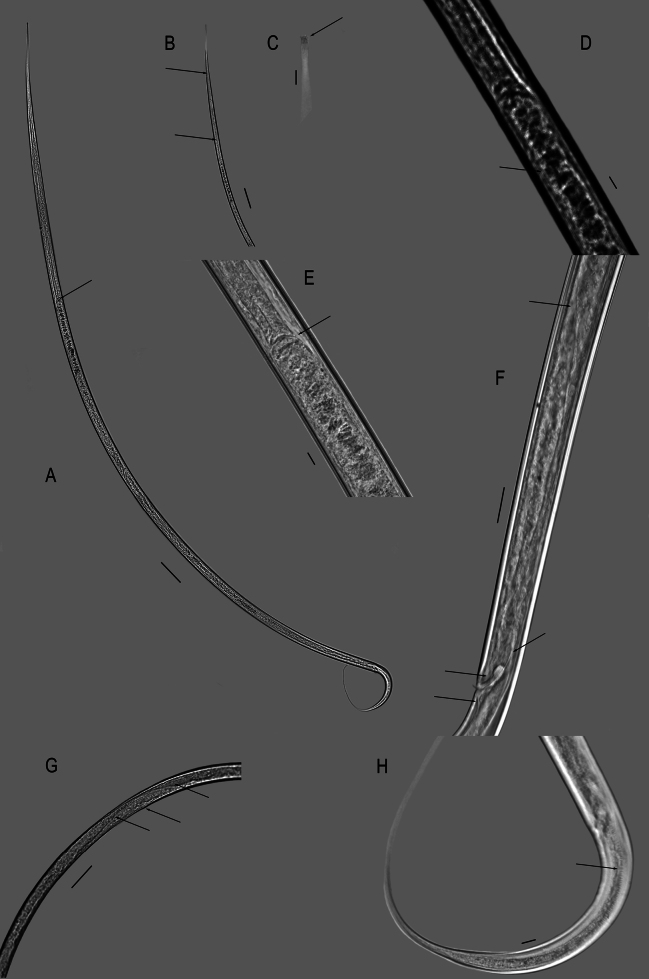
*Halalaimus pierri* sp. nov. Holotype male. **A**. Habitus, arrow pointing to the end of pharynx; **B**. Anterior end, arrows pointing to the long amphid; **C**. Arrow pointing to the setae in a zoom of the head; **D**. Arrow pointing to the lateral differentiation and punctate cuticle; **E**. Posterior end of the pharynx enlarged, arrow pointing to the cardia embedded in the intestine; **F**. Posterior end, arrows point to the end of the posterior outstretched testis, the spicules, the gubernaculum and the caudal apophysis; **H**. Male tail showing the ornamentation of the caudal alae, arrow pointing to the ornamentation of the caudal alae. Paratype female; **G**. Reproductive system, arrows pointing to the end of the reflexed ovaries and the vulva. Scale bar: 50 µm (**A**); 75 µm (**B**); 5 µm (**C, D, E, H**); 10 µm (**F**); 25 µm (**G**).

Figs 6, 7, Tables 1, 4

##### Type material.

***Holotype*** male (Inventory UGMD_104489). ***Paratypes*** 2 females (Inventory UGMD_1004490 and UGMD_1004491), are deposited in the voucher collection of the Gent University Museum Zoology Collections - UGent.

##### Type habitat and locality.

Specimens sampled in the CCZ. Deep sea sediment dominated by silt, containing polymetallic nodules. *Halalaimus pierri* sp. nov. was found in samples from two different expeditions to the GSR contract area in the CCZ, GSRNOD15A and GSRNOD17, in two stations: NodRich_C and NodRich_D in depths of ~ 4500 m, from 0–5 cm of sediment dominated by silt and clay particles.

##### Description.

Male holotype. Body slender, tapering at both extremities; anterior end very strongly tapering. Total length 1622 µm. Cuticle smooth and thin anteriorly, appears punctate from midbody to precloacal region, thickening from posterior third of amphidial region to mid-tail; maximum thickness 2.5 µm at cloacal level. Somatic and caudal lateral differentiation present; caudal lateral alae ornamented with fine striations. Cuticle pores not observed. Metanemes not observed. Anterior end narrow; head not set off. Inner labial papillae not observed. Six outer labial setae, 2 µm long; four cephalic setae, 1 µm long; OLS and CS separated by 1.5 µm. Amphideal fovea elongated, 85 µm long, 1.5 µm wide. Cardia embedded in intestine. Nerve ring located at 35% of pharyngeal length. Secretory-excretory system not observed. Reproductive system diorchic, with opposed and outstretched testes (anterior testis not very clear). Spicules paired, symmetrical, slender, curved, 21 µm long; velum visible. Gubernaculum 12 µm long, strongly sclerotised, curved, with anterior swelling and distinct caudal apophysis; enclosing laterally the posterior portion of spicules, tapering posteriorly. Precloacal sensillum absent. Caudal glands not observed. Tail conical-filiform; distal half filiform. Tail length 212 µm. Tail tip blunt. Spinneret not observed.

Females. Similar to male but shorter. Reproductive system didelphic, amphidelphic, reflexed ovaries. Spermatheca not seen. Vulva situated mid-body. Vagina perpendicular, not sclerotised. No vaginal glands observed. Caudal glands not observed. Spinneret not observed.

Juveniles. The two examined juveniles (*n* = 2) measured 1024 and 1240 µm in total length. The nerve ring was located 137 µm from the anterior end in both specimens, and the pharynx measured 291 and 315 µm, respectively. Body width values were mbd = 13–18 µm and abd = 8–10 µm, and the tail length ranged from 97 to 150 µm. The caudal alae were present, but ornamentation could not be visualised in juvenile specimens. Aside from reduced size, no additional deviations from the adult morphology were observed.

##### Diagnosis.

*Halalaimus pierri* sp. nov. is characterised by an elongated body, with a very pronounced anterior tapering. Body length ranges from 1492–1622 µm. Cuticle smooth anteriorly, showing punctations, from midbody to precloacal region, and somatic alae. Cuticle thickness increases from the posterior third of the amphid aperture and decreases again at mid-tail, just before the beginning of the filiform portion. Caudal alae present, ornamented, extending from just posterior to the cloaca to the onset of the filiform tail. Inner labial sensilla not observed; short outer labial sensilla setiform, 2–3 µm long; shorter cephalic setae 1–2 µm long only visible under high magnification; outer labial sensilla and cephalic setae separated by 1.5–2 µm. No somatic setae observed. Amphideal aperture 85 µm long and 1–1.5 µm wide. Pharynx elongated and muscular; cardia embedded in the intestine. Reproductive system diorchic, with opposed and outstretched testes, although the end of the anterior testis not clearly distinguished. Spicules curved, 21 µm long, 1.5 abd, with a visible velum. Gubernaculum strongly sclerotised, 12 µm long, curved, with an anterior swelling, tapering posteriorly, and bearing a weakly cuticularised caudal apophysis. Precloacal sensillum or cuticular pore absent. Caudal glands not distinguished. Tail elongated, with filiform tip. Based on the combination of absence of precloacal supplement and presence of caudal alae, *H. pierri* sp. nov. belongs to Group 2 of Keppner’s (1992) classification.

##### Differential diagnosis.

*Halalaimus pierri* sp. nov. is readily distinguished within Group 2 by its very long and slender anterior end; punctate cuticle; cephalic setae shorter than outer labial setae, both very short and not easily visible; the presence of an ornamented caudal alae; a strongly sclerotised, curved gubernaculum with a caudal apophysis, lacking a precloacal sensillum. The presence of a punctate cuticle in *H. pierri* sp. nov. is not easily observed and it is for the first time reported in Group 2. Until now, punctae have only been reported in Group 1 species: *H. bayensis*, *H. floridanus*, and *H. variabilis*; these species present vermiculations which are absent in *H. pierri* sp. nov. Also *H. dimorphus* has a punctate cuticle but the ILS are setae, whereas the ILS are invisible in *H. pierri* sp. nov. The new species shares an elongated amphid with *H. longamphidus* (amphid length: 85 µm in *H. pierri* sp. nov. vs. 74–81 µm in *H. longamphidus*), but differs significantly in body size of males (1622 µm vs. 2173–3391 µm respectively) and by the lack a bifurcate tail tip. It resembles *H. aciculus* by the abrupt tapering of the long anterior end and the presence of an ornamented caudal alae but differs in body length (1029–1064 µm in *H. aciculus* vs. 1492–1622 µm in *H. pierri* sp. nov.), a wider spacing between outer labial and cephalic setae (5.0–5.5 µm vs. 1.5–2 µm) and the absence of apophyses in *H. aciculus*, belonging to Group 1. Within Group 2, *H. pierri* sp. nov. is most similar to *H. lineatoides*, but can be distinguished by its significantly longer body, more pronounced anterior tapering, and distinct gubernaculum morphology, including the presence of a caudal apophysis—a feature rarely observed in other species (e.g., *H. gracilis*, *H. minusculus*, *H. sarsi*). These combined characters, including the absence of precloacal supplement, presence of ornamented caudal alae, and the unique gubernaculum structure—clearly differentiates *H. pierri* sp. nov. from all other described species within the genus. Among the deep sea species (deep sea key) it can be recognisable by somatic lateral alae absent and ornamented caudal alae present, gubernaculum with caudal apophysis and tail tip not bifurcate.

##### Etymology.

The new species is named after the first author’s husband, Pierre Misseghers.

### Keys

Keppner (1992) proposed four *Halalaimus* species identification keys for males and one for females. These keys were later updated: for Group 1 by Gagarin (2016) with a pictorial key; for Group 4 by Gagarin (2020); and for Group 2 by Shimada et al. (2020) and Leduc (2023). Gagarin and Nguyen Vu Thanh, (2018, in Russian) presented a key for nine Vietnamese species, and Huang and Zhang (2005) provided a table summarising the main distinguishing characters of *Halalaimus* species with a bifurcated tail tip and a key to 12 similar species.

In the present manuscript, we have updated these keys for the four *Halalaimus* groups by adding recently described species, including *H. sagarensis*, and *H. longamphidus*, which were previously not included. Additionally, we provide a new key for the identification of deep sea species, compiling all distinguishing features described so far for the 17 currently known deep sea species.

Because adult nematodes can nearly double in length during their lifetime—and because most species descriptions are based on a limited number of specimens—we avoided relying on absolute measurements. Instead, we use approximate ratios and visual comparisons, which are more user-friendly. For instance, sensilla length is expressed relative to head diameter; when these data were not provided, we inferred them from measurements and drawings. Note, however, that depending on the degree of anterior body attenuation, this approach can be challenging; therefore, we recommend comparing as many specimens as possible.

The keys focus primarily on the length and position of the anterior sensilla and their proportions relative to head diameter, along with distinctive features present only in a few species. The data are based on the morphometric table compiled for the species list: https://doi.org/10.5281/zenodo.19406582. When inconsistencies were found between original and subsequent redescriptions, we prioritised information from the original description, except in cases where later publications provided additional data (e.g., for the opposite sex).

#### Adapted key from Keppner (1992) for males of group 1

Note. Caudal alae present and precloacal supplement present.

Keppner (1992) provided a key for males of ten species belonging to Group 1. Since then five additional species have been described within this group: *H. aciculus*, *H. dimorphus*, *H. minimus*, *H. orientalis*, and *H. vietnamicus*. The latter three species were included in Gagarin (2016) updated key; however, although *H. aciculus* was classified by Gagarin and Nguyen Vu Thanh, (2014) as belonging to Group 1, it was not incorporated into that key. Here we present an updated version of the key for Group 1, including all relevant species described to-day, as well as *H. marcosi* sp. nov.

We omitted the presence/absence of the glandular structure connected to the pore as important distinguishing character because the visibility of the gland may depends upon the activity status of the gland.

**Table:** 

1	Precloacal supplement = pore	**2**
–	Precloacal supplement = sensillum	**3**
2	Precloacal pore present; somatic setae short and spiny; OLS = CS = 1 HD long; ornamented caudal alae and vermiculations absent	***H. sobakini***
–	Precloacal pore present; somatic setae absent but cuticular pits present; OLS = CS ~ 3 HD long; ornamented caudal alae and vermiculations present	***H. variabilis***
3	Inner labial sensilla = setae	**4**
–	Inner labial sensilla = papillae	**5**
–	Inner labial sensilla not visible	**9**
4	Cuticle with transverse rows of small dots; OLS = CS = 1.6 HD long and 1.5 HD apart; sperm dimorphism in testes	***H. dimorphus***
–	Cuticle with transverse striations without dots; OLS < CS at ~ 1 HD apart, no sperm dimorphism	***H. tarjani***
5	OLS < CS respectively ~ 0.8–1 HD long and ~ 0.8 HD apart; somatic setae at level of amphid; somatic alae and non-ornamented caudal alae present	***H. marcosi* sp. nov**.
–	OLS > CS respectively 1.8–1.1 HD long and 0.5 HD apart; somatic setae absent; somatic alae absent, ornamented caudal alae present	***H. orientalis***
–	OLS = CS	**6**
6	OLS & CS > 1 HD long	**7**
–	OLS & CS ≤ 1 HD long	**8**
7	Length OLS & CS ~ 1.5 HD long and at 0.8 HD apart from each other; somatic setae absent	***H. paracomatus***
–	Length OLS & CS ~ 2.5 HD long and at 1 HD apart from each other; cervical, somatic and caudal cervical setae present	***H. thalassinus***
–	Length OLS & CS ~ 3 HD long and at 0.4–0.5 HD apart from each other; somatic setae absent	***H. bayensis***
8	Length OLS & CS ~ 1 HD long and well separated at 1.2 HD apart	***H. bulbocaudatus***
–	Length OLS & CS < 1 HD long and very well separated at 3.5–4 HD apart	***H. vietnamicus***
9	OLS > CS respectively at 1.4–1.2 HD long and close together at 0.2 HD apart	***H. comatus***
–	OLS < CS	**10**
–	OLS = CS	**11**
10	OLS < CS respectively at 1 and 1.2 HD long and –2.5 HD apart; length amphid ~ 10 HD; vermiculations absent	***H. aciculus***
–	OLS < CS respectively at 1.1 and 1.6–2 HD long and about 2 HD apart; length amphid ~ 17–20 HD vermiculations present	***H. americanus***
11	OLS & CS very long ~ 4.5 HD long and ~ 1 HD apart; caudal alae ornamented	***H. floridanus***
–	OLS & CS < 1 HD long and about 0.8 HD apart; caudal alae not ornamented	***H. minimus***

#### Adapted key from Keppner (1992) for males of group 2

Note. Caudal alae present, precloacal supplement absent.

Keppner (1992) provided a key for the males of nine species belonging to Group 2. Since then six additional species have been described within this group: *H. gidanensis*, *H. durus*, *H. minor*, *H. longamphidus*, *H. shinkai*, and *H. talaurinus*. Updated keys were provided by Shimada et al. (2020) and later adopted by Leduc (2023), incorporating most of these new species. However, *H. longamphidus* was not included in either version (probably because in the original description the presence of ornamented caudal alae is not mentioned, but is clearly drawn in his figure 4). Additionally in both keys *H. durus* is erroneously reported as having papilliform inner labial sensilla, while in fact “Labial рарillае not seen” is written. In the updated key presented below, both *H. longamphidus* and *H. pierri* sp. nov. are included.

**Table:** 

1	Tail tip bifurcate	**2**
–	Tail tip not bifurcate	**3**
2	Inner labial sensilla setae; head constriction absent; OLS < CS; very long amphid	***H. longamphidus***
–	Inner labial sensilla invisible, OLS > CS; head constriction present	***H. brimi***
3	Gubernaculum with apophysis	**4**
–	Gubernaculum without apophysis	**7**
4	OLS > CS; very markedly tapering of the anterior end; length amphid ~ 20 HD	***H. pierri* sp. nov**.
–	OLS = CS	**5**
5	Head labial capsule present; OLS = CS 2 HD long	***H. sarsi***
–	Head capsule absent; OLS & CS shorter	**6**
6	OLS & CS very short 0.2 HD long at distance of 0.5 HD apart; somatic alae cross striated; tail with 3 caudal papillae; small size (504–569 µm)	***H. minor***
–	OLS & CS ~1.5 HD long at distance of 1 HD apart; cross striation absent; very thin body (a = ~ 130)	***H. gerlachi***
7	ILS = setae, length 0.25 HD; OLS ≥ CS; somatic alae present	***H. gracilis***
–	ILS = papillae; OLS < CS	**8**
–	ILS invisible	**9**
8	Cuticle very thick (≥ 40% cbd) with 8 longitudinal rows of pores; head constriction present; OLS < CS, both ~ 0.5 HD and very close (0.2 HD); long body (~ 3 mm)	***H. talaurinus***
–	Cuticle longitudinally striated; OLS < CS, both very short ≤ 0.3 HD and ~ 1 HD apart	***H. gidanensis***
–	Cuticle smooth; OLS < CS, respectively 0.3–1 HD and ~1.2 HD apart	***H. alatus***
9	OLS > CS both > 1.5× HD and very close to each other; tail conical - filiform	***H. relatus***
–	OLS < CS	**10**
–	OLS = CS	**11**
10	OLS < CS, OLS setiform papillae; both circles at 0.5 HD apart; cuticle with 8 rows of setae; spicules ~ 2 abd; long body (~ 4 mm)	***H. shinkai***
–	OLS < CS, OLS setae 0.3 HD at 1 HD apart; cuticle clearly sclerotised without rows of setae	***H. lineatoides***
11	Cuticle without somatic lateral alae; OLS & CS ~ 2.5 HD at 1.2 HD apart; c' = 15–20; spic 1.5 abd	***H. durus***
–	Cuticle with somatic lateral alae	**12**
12	OLS = CS ~ 1 HD long at 1 HD distance; bright somatic lateral alae present; tail conical-cylindrical c' = 13	***H. lineatus***
–	OLS & CS ~ 1.3 HD, at 1 HD distance; tail very long, flagelliform, c' = 42	***H. filum***

#### Adapted key from Keppner (1992) for males of group 3

Note. Caudal alae absent, precloacal supplement present.

After Keppner (1992) only one new species has been described: *H. dolgovi*. *Halalaimus longicaudatus* was transferred to Group 3 because Filipjev (1929) noted that a very small precloacal papilla was present (as well as a postcloacal flattening). Currently the group consist of 14 species.

**Table:** 

1	Precloacal pore/papilla present, sensillum absent	**2**
–	Precloacal sensillum present, pore absent	**3**
2	Postcloacal flattening present; gubernacular apophysis absent	***H. longicaudatus***
–	Postcloacal flattening absent; gubernacular apophysis present	***H. cubanus***
3	Tail tip bifurcate; ILS setiform	***H. parafletcheri***
–	Tail tip not bifurcate	**4**
4	ILS setiform; OLS & CS equal in length; tail tip swollen	***H. curvicaudatus***
–	ILS papillae or not discernible/visible	**5**
5	OLS > CS	**6**
–	OLS = CS	**7**
–	OLS < CS	**10**
6	OLS very long, length 3 HD and 3 CS; c'= 9	***H. horridus***
–	OLS very long, length 3.5 HD and 1/3 > CS; c'=14	***H. striatus***
7	OLS & CS circles close to each other (< 1 HD)	**8**
–	OLS & CS circles well separated (≥ 1 HD)	**9**
8	Length OLS = CS > 1HD; tail very long (c'= 106) with very long filiform part	***H. monstrocaudatus***
–	Length OLS = CS < 1HD; tail not so very long (c'=14–15) filiform part 50%	***H. terrestris***
9	OLS & CS length 2 HD at ~ 1 HD apart from each other, sexual dimorphism in amphid length	***H. nigrilapidarius***
–	OLS & CS length 2–4 HD at 1.5 HD apart from each other	***H. cirrhatus***
10	Gubernaculum with dorsocaudal apophysis	***H. minusculus***
–	Gubernaculum without apophysis	**11**
11	Somatic setae present along the amphid	***H. delamarei***
–	Somatic setae absent	**12**
12	Somatic alae present, caudal alae absent; 2 precloacal setae and spermatheca present; freshwater	***H. dolgovi***
–	Somatic alae absent, caudal alae present, 1 precloacal seta; marine	***H. marri***

#### Adapted key from Keppner (1992) for males of group 4

Note. Caudal alae absent, precloacal supplements absent.

The original key for Group 4 by Keppner (1992) included 30 species. Gagarin (2020) provided an updated version, incorporating *H. borealis* sp. nov. (here referred as *H. gagarini* nom. nov.), *H. longipharynx*, and *H. parvulus*, but omitted *H. luticolus*. In the updated key presented below, we have added the species described by Bussau (1993): *H. abyssus*, *H. aedificandistudiosus*, *H. oblongus* as well as *H. luticolus* and *H. sagarensis*. *Halalaimus ciliocaudatus and H. longistriatus* were excluded as they were described from a female specimen only (see also Shimada et al. 2020). *Halalaimus longicaudatus* was transferred to Group 3 because Filipjev (1929) noted that a very small precloacal papilla was present (as well as a postcloacal flattening).

**Table:** 

1	Head without anterior sensilla	**2**
–	Head with anterior sensilla	**3**
2	Cuticle thin; tail with conical and cylindrical part in equal proportion; gubernaculum absent	***H. leptoderma***
–	Cuticle very thick; tail with long conical part (5/6) and short cylindrical part (1/6); gubernaculum present but weakly developed	***H. pachyderma***
3	Head with ILS papillae and only 6 OLS	***H. sagarensis***
–	Head with ILS = setae, 6 OLS and 6 CS; tail tip bifurcate	***H. filicollis***
–	Head with only 4 CS	**4**
–	Head with 6 OLS and 4 CS	**5**
4	Length CS 0.7 HD; amphid invisible; body very long (L > 5 mm) and very thin (a = ~ 200)	***H. leptosoma***
–	Length CS 1.8 HD; amphid distinct; body medium size & shape (L ~ 2.5 mm, a = 86)	***H. brevispiculum***
5	Tail tip bifurcate	**6**
–	Tail tip not bifurcate	**7**
6	OLS > CS, both > HD, at ~0.5 HD apart from each other	***H. aedificandistudiosus***
–	OLS < CS, both > HD; ILS = setae	***H. fletcheri***
–	OLS = CS	***H. diacros***
7	ILS = setae; OLS < CS both little longer than HD and close together	***H. jaltensis***
–	ILS papillae	**8**
–	ILS invisible	**9**
8	OLS = CS; head capsule present	***H. papillifer***
–	OLS < CS; head capsule absent	***H. parvulus***
9	6 OLS + 6 CS; length OLS = CS	***H. setosus***
–	6 OLS + 4 CS	**10**
10	Gubernaculum absent	**11**
–	Gubernaculum present	**12**
11	OLS ≥ CS and 5 HD long; head constriction absent	***H. supercirrhatus***
–	OLS > CS, close together; head constriction present	***H. turbidus***
–	OLS = CS, 1 HD long and close together; head constriction present	***H. macquariensis***
–	OLS = CS, 2 HD long, in 1 circle; head constriction absent	***H. rectispiculatus***
12	OLS > CS at more than 1 HD apart; somatic lateral alae present	***H. zenkevitshi***
–	OLS < CS	**13**
–	OLS = CS	**16**
13	Gubernaculum with apophysis; OLS & CS very long at 1 HD apart; length CS 4 HD; body very thin (a = 150)	***H. florescens***
–	Gubernaculum without apophysis	**14**
14	Only CS > 1 HD; cuticular pores present	***H. abyssus***
–	CS = 1 HD, cuticular pores absent	***H. longipharynx***
–	Both OLS & CS > 1 HD	**15**
15	CS = 2 HD; cuticle striated with pores; tail conical-filiform with short (1/6) conical part	***H. oblongus***
–	CS < 1 HD; cuticle smooth, pores absent; tail conical-cylindrical with long (3/5) conical part	***H. wodjanizkii***
–	OLS = CS	**16**
16	Length OLS & CS < 1 HD = 0.2 HD, well separated at > 1 HD apart; somatic lateral alae present	***H. isaitshikovi***
–	Length OLS & CS 4–6 HD, ~ 1 HD apart, very thin body (a = ~ 150)	**17**
–	Length OLS & CS ~ 2 HD	**18**
–	Length OLS & CS slightly > 1 HD	**19**
–	Length OLS & CS = 1 HD	**20**
17	Tail very long (c = 5, c’ = 43), conical-filiform with short conical part	***H. meyersi***
–	Tail shorter (c = 10, c’ = 15–20), conical-cylindrical	***H. capitulatus***
18	OLS and CS at 0.5× HD apart; head constricted; body very thin (a = ~ 200); tail conical-filiform	***H. filicorpus***
–	OLS and CS very close: 0.1 HD apart; head not constricted; medium body (a = 66); tail conical-elongated	***H. anne***
19	OLS & CS ~ 1 HD apart; length amphid 15 HD	***H. luticolus***
–	OLS & CS ~ 0.3 HD apart; length amphid 8 HD	***H. gagarini* nom. nov**.
20	Long and wide amphid ~ 40% cbd; OLS & CS close at 0.4 HD	***H. pachyodoroides***
–	Width amphid < 25% cbd	**21**
21	Tail conical-filiform	**22**
–	Tail conical-cylindrical	**23**
22	OLS & CS very close at 0.2 HD; very long amphid: 27 HD; conical part tail < 50%	***H. lutarus***
–	OLS & CS at 0.6 HD apart; conical part tail ~ 50%	***H. longicollis***
23	OLS & CS at 1 HD apart; length amphid = 3 HD	***H. parvus***
–	OLS & CS closer at 0.5 HD; length amphid = 6 HD	***H. caroliniensis***

#### Key to deep-sea species

As for some of the deep-sea species only females are described, this key is developed to be used in these cases as well; significant male characteristics are added where possible.

**Table:** 

1	Tail tip bifurcate	***H. aedificandistudiosus***
–	Tail tip not bifurcate	**2**
2	ILS = setae; head without constriction; OLS ≥ CS; precloacal sensillum absent	***H. gracilis***
–	ILS = papillae	**3**
–	ILS invisible	**4**
3	Head with constriction; lateral caudal alae not ornamented; precloacal sensillum present	***H. marcosi* sp. nov**.
–	Cuticle extremely thick (40–52% cbd); lateral caudal alae ornamented; precloacal sensillum absent	***H. talaurinus***
4	Gubernaculum with apophysis; precloacal sensillum absent, lateral somatic alae absent, caudal alae ornamented; OLS > CS, both < 1 HD, at ~ 1 HD apart	***H. pierri* sp. nov**.
–	Gubernaculum without apophysis	**5**
5	OLS & CS papilliform; cuticle with 8 rows of somatic setae; lateral somatic and ornamented caudal alae present; precloacal sensillum absent; long body (~ 4 mm)	***H. shinkai***
–	OLS & CS setiform	**6**
6	OLS = CS	**7**
–	OLS > CS	**8**
–	OLS < CS	**9**
7	OLS = CS, both 1.7 HD long, at ~ 0.5 HD apart; tail conical-cylindrical, striated; c' = 25	***H. filicorpus***
–	OLS = CS, both ~ 0.8 HD, at ≤ 1 HD apart; tail conical-cylindrical, tail tip spherical swollen; c' = 12	***H. longinquus***
8	OLS > CS, both < 1 HD, at > 1 HD apart; somatic lateral alae absent, caudal alae absent; precloacal sensillum absent; tail conical-filiform, short filiform part; c' = 14	***H. absconditus***
–	OLS > CS, both ≥ 1 HD, close together; very long amphid (~ 30 HD); tail conical-filiform, long filiform part; c' = 30	***H. egregius***
9	Length OLS < CS, respectively ~ 0.6 < 1.2 × 1 HD, at ~ 0.8 HD apart; cuticle with short setae along the sides of the amphideal fovea; tail conical-cylindrical; c' = 10	***H. delamarei***
–	Length OLS & CS respectively ~ 0.8 < 1 HD, at distance ~ 0.5 HD apart; tail conical-filiform, short conical part; c' = 28	***H. arundinaceus***
–	Length OLS & CS respectively ~ 1 < 1.5 HD, at distance of ~ 0.5 HD apart; tail conical-cylindrical, both parts equal, tail tip spherical; c' = ~ 14	***H. abyssus***
–	Length OLS & CS respectively ~ 1.6 < 2.2 HD and at ~ 0.8 HD apart; tail conical-filiform, short conical part; c' = 17	***H. oblongus***
–	Length OLS & CS respectively ~ 2 < 2.3× HD, at ~ 1.3 HD apart; tail conical-filiform, short conical part; c' = 23	***H. praestans***

#### Adapted key for all described species, including females

In this key we tried to avoid precise measurements, but used ratios which can be interpreted by eye, and focused on the anterior sensilla of the head. Although sometimes setae can be broken, in view of the number of setae it is rare they are all absent and at least the basis of the setae can be seen. This key is mainly based upon original descriptions (except when the other gender is described later). Keppner (1992) provided a key for females of 54 species; this updated key comprises 102 species.

**Table:** 

1	All anterior sensilla absent	**2**
–	Only 1 row of 4 or 6 setae present	**3**
–	6 Outer labial and 4 cephalic sensilla present	**4**
–	ILS = papillae and only 6 OLS = setae present	***H. sagarensis***
2	Cuticle very thick (25% cbd); tail conical-cylindrical, cylindrical part 20%	***H. pachyderma***
–	Cuticle not so thick; tail conical-cylindrical, cylindrical part slightly shorter than conical part	***H. leptoderma***
3	Only 1 row of 4 submedian CS; cuticle very thick; lateral alae broad (≥ 1/3 cbd); amphid absent; L > 5 mm; tail conical-cylindrical, swollen tip	***H. leptosoma***
–	Only 1 row of 4 or 6 setae present; lateral alae present; amphid absent; L < 2 mm; tail conical-cylindrical, tip spherical	***H. borealis***
–	Only 1 row of 4 or 6 setae; lateral alae absent; amphid present; tail conical-cylindrical, cylindrical part 33%	***H. brevispiculum***
4	Cuticle with longitudinal lines (different from lateral alae)	**5**
–	Cuticle without longitudinal lines	**6**
5	Cuticle with longitudinal striations; ILS & OLS papilliform; CS very short setae; tail conical-cylindrical, tip slightly swollen	***H. gidanensis***
–	Cuticle with ~ 36 longitudinal lines; OLS & CS setiform, OLS > CS and both < 1 HD; tail conical-cylindrical, cylindrical part ~60%, tip not swollen	***H. longistriatus***
6	Tail tip bifurcate	**7**
–	Tail tip not bifurcate	**11**
7	ILS = setiform	**8**
–	ILS not visible	**10**
8	Exceptional 6 CS present; length OLS = CS and < 1 HD, very close to each other	***H. filicollis***
–	Normal pattern of 4 CS	**9**
9	Length OLS < CS, respectively 1.2 < 1.8 HD and ~ 0.8 HD separated from each other; amphid length ~ 6 HD; c' ~ 12	***H. fletcheri***
–	Length OLS < CS, respectively 1 < 1.2 HD and ~ 0.5 HD separated from each other; amphid length ~ 7 hd; c' ~ 15	***H. parafletcheri***
–	Length OLS < CS, respectively 1.3 < 1.8 and close ~ 0.3 HD from each other; long amphid ~ 13 HD, anterior at ~ 4 HD; c' 14–16	***H. longamphidus***
10	Length OLS = CS = 1 HD and very close (0.1–0.2 HD) to each other	***H. diacros***
–	Length OLS slightly > 2 CS, respectively 1.5 > 0.7 HD, at 0.6 HD from each other; a ~ 100	***H. brimi***
–	Length OLS little < 2 CS, respectively 1.7 > 1.2 HD, at 0.6 HD from each other; a ~ 50	***H. aedificandistudiosus***
11	Inner labial sensilla discernible as papillae or setae	**12**
–	Inner labial sensilla not discernible	**23**
12	Inner labial sensilla setae	**13**
–	Inner labial sensilla papillae	**15**
13	OLS > CS, respectively 0.9 > 0.8 HD, at distance ~ 1 HD; somatic lateral alae present	***H. gracilis***
–	OLS < CS, respectively 0.5 > 0.9 HD and well separated (≥ 1 HD from each other); amphid broad, anterior at level of CS	***H. tarjani***
–	OLS = CS	**14**
14	Length OLS & CS = 0.9 HD, at 0.2 HD from each other; cuticle smooth, 3–5 pores in the amphideal fovea region; tail conical-filiform, tip globular	***H. deseadensis***
–	Length OLS & CS = 1 HD, at 0.1 HD from each other; cuticle thick and striated; tail conical-cylindrical, striated with a bent at transition conical-cylindrical part	***H. curvicaudatus***
–	Length OLS & CS = 1.6 HD, at 1.5 HD from each other; cuticle with transverse rows of dots; sperm & egg dimorphism; tail conical-cylindrical	***H. dimorphus***
–	Length OLS & CS ~ 1.3 HD, close together at 0.2 HD; tail conical-cylindrical, tip swollen; ILS = setiform papillae, head capsule present	***H. papillifer***
15	Cuticle with 4 or 8 rows of pores or setae and somatic lateral alae	**16**
–	Cuticle without pores or setae	**17**
16	Cuticle with 8 longitudinal rows of pores and very thick = 14–18 µm (40–52% cbd); OLS < CS both < 1 HD and very close	***H. talaurinus***
–	Cuticle with 4 longitudinal rows of setae and papillae; OLS = CS and ~ 2.5 HD, at 1 HD apart	***H. thalassinus***
17	OLS > CS, respectively 1.8 > 1.1 HD; at a distance of 0.6 HD apart; no lateral alae	***H. orientalis***
–	OLS < CS	**18**
–	OLS = CS	**20**
18	CS ~ 2 HD; OLS < CS respectively 0.8 < 2 HD long and > 1.2 HD separated	***H. parvulus***
–	OLS & CS ≤ 1 HD	**19**
19	OLS & CS respectively 0.8 < 1 HD long and ~ 1 HD separated; head constriction and somatic lateral alae present; tail conical-filiform, filiform part 50%, tip fine	***H. marcosi* sp. nov**.
–	OLS & CS respectively 0.3 < 1 HD long and > 1.2 HD separated; somatic lateral alae absent; tail conical-cylindrical; cylindrical part 50%, tip clavate	***H. alatus***
–	OLS < CS respectively 0.7 < 1 HD long and about 1 HD separated; somatic lateral alae absent; tail conical-cylindrical; cylindrical part ~ 46%, tip swollen; freshwater species	***H. algeriensis***
20	OLS = CS; cuticle with transverse striae and punctations at mid body + pits; somatic lateral alae absent; OLS & CS well separated (~ 1 HD); tail conical-cylindrical, cylindrical part with coarse striations	***H. bayensis***
–	OLS = CS; cuticle without punctations and pits	**21**
21	Somatic lateral alae present; length OLS = CS > 1 HD (1.3–1.6 HD) at ~ 0.3 HD apart; tail conical-cylindrical, cylindrical part with coarse striations, tip blunt, slightly bulbous	***H. paracomatus***
–	Somatic lateral alae absent	**22**
22	OLS = CS > 1 HD (1.1 HD), at 0.9 HD from each other; tail conical-cylindrical, conical > cylindrical part, latter without striations, tip spherically swollen	***.H. bulbocaudatus***
–	OLS = CS < 1 HD (0.7–0.8 HD), at 0.6 HD from each other; tail conical-cylindrical, cylindrical part 32–38%, tip club-shaped & swollen	***H. vietnamicus***
23	Somatic lateral alae present	**24**
–	Somatic lateral alae absent	**29**
24	Somatic lateral alae cross striated; OLS = CS = 0.2 HD at 1 HD apart; tail conical-filiform, cylindrical portion 66%, tip swollen + spinneret	***H. minor***
–	Somatic lateral alae not cross striated	**25**
25	OLS = CS	**26**
–	OLS < CS	**27**
–	OLS > CS	**28**
26	OLS = CS = 0.5 HD at 0.8 HD apart; cuticle smooth; tail conical-cylindrical, cylindrical part 33%, tip swollen	***H. isaitshikovi***
–	OLS = CS = 0.8–1.3 HD at 1 HD apart; cuticle finely striated; tail very long (c' = 42), conical-filiform, filiform part ± 75%, tip cylindrical	***H. filum***
–	OLS = CS = 1 HD at 1 HD apart; cuticle striated; tail conical-filiform, filiform part 54%, tip bluntly rounded	***H. lineatus***
–	OLS = CS = 1 HD and very close (0.1 HD); cuticle thick (1/6 cbd), smooth; tail conical-cylindrical, cylindrical part 25%	***H. pachydermatus***
–	OLS = CS = 1.5 HD at 1 HD apart; cuticle smooth; gubernacular apophysis present, tail conical- cylindrical, c' = 23–35, tip swollen	***H. gerlachi***
–	OLS = CS = 2 HD at 0.7 HD apart; cuticle thick; tail short (c' = 9), conical-cylindrical, cylindrical part ± 50%, tip conical + spinneret	***H. cubanus***
27	OLS = papillae; CS very short 0.3 HD; cuticle sclerotized; lateral alae 1/10 cbd, tail conical-filiform, 75% filiform, tip fine	***H. lineatoides***
–	Length OLS < CS, respectively 1 < 1.2 HD and far apart (2.5 HD); cuticle striated; tail conical-cylindrical, cylindrical part ± 40%	***H. aciculus***
–	Length OLS < CS, respectively 1 < 1.8 HD and far apart (2 HD); cuticle striated; lateral alae broad; tail conical-cylindrical, cylindrical part ± 40%, coarsely striated, tip blunt	***H. americanus***
–	Length OLS < CS, respectively 1 < 1.3 HD and far apart (1.5 HD); cuticle smooth; head capsule present; amphideal fovea broad = 1/3 cbd; tail conical-cylindrical, cylindrical part 50%, tip swollen	***H. dolgovi***
28	OLS > CS, respectively 1.7 > 1.5 HD and very close (0.2 HD); tail conical-filiform, filiform part ~ 80%	***H. relatus***
–	OLS > CS, respectively 1 > 1 HD and well separated (1.4 HD); tail conical-cylindrical, cylindrical part 20–40%	***H. zenkevitshi***
29	OLS < CS	**30**
–	OLS > CS	**36**
–	OLS = CS	**40**
30	OLS < CS, both > 2 HD	**31**
–	OLS < CS, length of both > 1 ≤ 2 HD	**32**
–	OLS < CS, length of both ≤ 1 HD	**33**
31	CS 4 HD; OLS & CS at 1 HD apart; cuticle striated; tail conical-cylindrical, cylindrical part 67%, tip blunt, c' = ~ 17	***H. florescens***
–	CS 2.2 HD; OLS & CS at 0.5 HD apart; cuticle smooth; tail conical-cylindrical, cylindrical part 33%, tip pointed, c' = ~ 11–17	***H. marri***
–	CS 2.3 HD; OLS & CS at 1.3 HD apart; cuticle striated; tail conical-filiform, filiform part 80%, tip fine, c' = ~ 23	***H. praestans***
32	OLS & CS respectively 1.3 < 1.8 HD, well separated at ~ 1.5 HD apart; head off set; tail conical-cylindrical, cylindrical part 25%, tip swollen; c' = 9	***H. jaltensis***
–	OLS & CS respectively 1 < 1.5 HD, close at ~ 0.5 HD apart; cuticle with pores; tail conical-cylindrical, both parts ~ 50%, tip swollen; c' = 14	***H. abyssus***
–	OLS & CS respectively 1.3 < 1.6 HD, close at ~ 0.8 HD apart; cuticle with small pores; tail conical-filiform, filiform part 80%, tip fine; c' = 17	***H. oblongus***
–	OLS & CS respectively 1 < 1.3 HD, close at ~ 3 HD apart; tail conical-cylindrical, cylindrical part 40%, tip swollen; c' = 14	***H. stammeri***
–	OLS & CS respectively 1.3 < 1.6 HD, close at ~ 0.8 HD apart; tail conical-cylindrical, cylindrical part 40%, tip rounded; c' = 7–12	***H. wodjanizkii***
33	OLS & CS respectively 0.2 < 0.4 HD, well separated at ~ 1 HD apart; amphid wide, anterior end at level CS; tail conical-filiform, filiform part 30%, tip swollen, c' = 8	***H. climactericus***
–	OLS & CS respectively 0.5 < 1 HD, at ~ 0.7 apart; tail conical-cylindrical, cylindrical part 35%, tip swollen	***H. longipharynx***
–	OLS & CS respectively 0.6 < 1 HD, well separated at ~ 1 HD apart; tail conical-cylindrical, cylindrical part 25%, tip not swollen, c' = 12–15	***H. minusculus***
–	OLS & CS respectively 0.6 < 1.2 HD, at ~ 0.8 HD apart; cervical and caudal papilliform setae present; tail conical-cylindrical, cylindrical part 25%, tip swollen, c' = 9–10	***H. delamarei***
–	OLS & CS respectively 0.7 < 1 HD, well separated at 1 HD apart; cuticle duplicated at the head; tail conical-filiform, cylindrical part 25%, tip fine, c' = 10–14	***H. diplocephalus***
–	OLS & CS respectively 0.8 < 1 HD, close at ~ 0.5 HD apart; tail conical-filiform, filiform part 60%, tip fine, c' = 16	***H. arundinaceus***
–	OLS & CS respectively 0.9 < 1 HD, well separated at 1 HD apart; tail conical-filiform, filiform part ~ 70%, tip fine, c' = 16–23	***H. amphidellus***
34	OLS > CS, respectively 1.3 > 0.7 HD, very close at 0.3 HD; tail conical- filiform, filiform part 50–75%, c' = 12–16; sexual differences in length setae, amphid & tail	***H. turbidus***
–	OLS > CS, respectively 3 > 1 HD and 0.6 HD apart; tail conical-cylindrical, cylindrical part 50%, tip with knob-like swelling; c' = 9	***H. horridus***
–	OLS > CS, both ≥ 2 HD long	**35**
–	OLS > CS, both ≥ 1 ≤ 2 HD long	**36**
–	OLS > CS, both < 1 HD long	**37**
35	OLS > CS, respectively 5 > 3 HD and 0.8 HD apart; cuticle striated and punctated; tail conical-cylindrical, cylindrical part 50%, c' = 14	***H. longisetosus***
–	OLS > CS, respectively 3.5 > 3 HD and 0.6 HD apart; cuticle striated; tail conical-cylindrical, cylindrical part 50%, c' = 14	***H. striatus***
–	OLS > CS, respectively 3 > 2 HD and well separated at 1.5 HD apart; cuticle thickly sclerotized; tail striated, conical-cylindrical, tip swollen	***H. scleratus***
36	OLS > CS, respectively 1.4 > 1.2 HD and 0.3 HD apart; cuticle striated; tail conical-cylindrical, cylindrical part 25%, tip with knob-like swelling; c' = 10–15	***H. comatus***
–	OLS > CS, respectively 1.3 > 1 HD and 0.2 HD apart; cuticle with small pores; amphid very long: 29 HD; tail conical-filiform, filiform part ~ 84%; c' = 30	***H. egregius***
–	OLS > CS, respectively 1.2 > 1 HD and 0.5 HD apart; amphideal fovea close to CS; tail uniformly tapering; c' = 15	***H. luticolus***
37	OLS > CS, respectively 0.8 > 0.5 HD, well separated at 1.3 HD apart; head capsule present; tail conical-filiform, filiform part 25%, spinneret distinct	***H. absconditus***
–	OLS > CS, respectively 0.8 > 0.5 HD and 1 HD apart; tail conical- filiform, filiform part 60%, tip blunt, spinneret invisible	***H. pierri* sp. nov**.
–	OLS > CS, respectively 0.8 > 0.5 HD, very close at 0.2 HD apart; tail conical- filiform, filiform part 50%, tip fine	***H. longicaudatus***
38	OLS = CS very long > 2 HD	**39**
–	OLS = CS > 1 ≤ 2 HD	**40**
–	OLS = CS = 1 HD	**41**
–	OLS = CS < 1 HD	**42**
39	OLS & CS = 5 HD long, well separated at 4 HD apart; tail conical-cylindrical, cylindrical part 50%, tip swollen	***H. supercirrhatus***
–	OLS & CS = 4–6 HD long, at 1 HD apart; tail conical-cylindrical, cylindrical part 66;% tip blunt	***H. capitulatus***
–	OLS & CS = 4.3 HD long, well separated at 1.5 HD apart; tail conical-cylindrical, cylindrical part 33%, tip barely swollen	***H. cirrhatus***
–	OLS & CS = 4.5 HD long, at 1 HD apart; cuticle striated and punctated; tail conical-cylindrical, cylindrical part ~ 50% with coarse striations, tip blunt with spinneret	***H. floridanus***
–	OLS & CS = 4.4 HD long, at 1 HD apart; tail very long and thin, conical-filiform, filiform part ~ 85%, tip narrow	***H. meyersi***
–	OLS & CS = 2.8–4 HD long, at 1 HD apart; cuticle striated and punctated; tail conical-cylindrical cylindrical part with coarse striae, cylindrical part ~ 50%, tip narrow with spinneret	***H. variabilis***
–	OLS & CS = 2.2–3 HD long, close at 0.5 HD apart; cuticle smooth except tail; tail conical-filiform, filiform part ~ 90% and striated; c' = 106	***H. monstrocaudatus***
–	OLS & CS = 3 HD long, close at 0.6 HD apart; head capsule present; tail conical-cylindrical cylindrical part 50%, tip blunt	***H. sarsi***
–	OLS & CS = 2.5 HD long, well separated at 1.2 HD apart; tail conical-cylindrical, cylindrical part 50%, tip swollen	***H. durus***
40	OLS & CS = 1.7 HD long, well separated at 1.3 HD apart; exceptionally 6 cephalic setae present; tail conical-filiform, filiform part 65%	***H. setosus***
–	OLS & CS = 2 HD long, separated at 1 HD apart; body very thin: a = 213; head constriction present; tail conical-cylindrical, cylindrical part 39–48%, tip swollen, sexual dimorphism in amphideal fovea	***H. nigrilapidarius***
–	OLS & CS = 2 HD long, very close in 1 circle; tail conical-cylindrical, cylindrical part 30%, tip swollen	***H. rectispiculatus***
–	OLS & CS = 1.7 HD long, close at 0.5 HD apart; conical-filiform, striated, filiform part 75%, tip truncated	***H. filicorpus***
–	OLS & CS = 1.7 HD long, very close at 0.1 HD apart; tail conical-filiform, filiform part ~ 50%, tip pointed	***H. anne***
–	OLS & CS = 1.2 HD long, close at 0.3 HD apart; tail conical-filiform, filiform part 66–70%, tip pointed	***H. gagarini* nom. nov**.
–	OLS & CS = 1.1 HD long, close at 0.4 HD apart; tail conical-filiform, filiform part 66–80%	***H. pachyodoroides***
41	OLS & CS = 1 HD long, close at 1.2 HD apart; cuticle striated; head constriction present; tail conical-filiform, filiform part 13%, tip fine	***H. macquariensis***
–	OLS & CS = 1 HD long, close at 0.6 HD apart; tail conical-cylindrical, conical part striated, cylindrical part 60%, tip swollen	***H. amphistrius***
–	OLS & CS = 1 HD long, close at 0.5 HD apart; tail conical-cylindrical, smooth, gradually tapering, cylindrical part short, tip swollen	***H. caroliniensis***
–	OLS & CS = 1 HD long, close at 0.6–0.8 HD apart; tail conical-filiform, filiform part ~ 50%, tip fine, dorsally bent	***H. longicollis***
–	OLS & CS = 1 HD long, at 1 HD apart; tail conical-cylindrical, cylindrical part 50%, tip swollen	***H. parvus***
–	OLS & CS = 1 HD long, very close at 0.2 HD apart; long amphid; tail conical- filiform, filiform part part 60%, tip fine	***H. lutarus***
–	OLS & CS = 1 HD long, very close at 0.2 HD apart; cuticle clearly striated; tail conical-cylindrical, cylindrical part 20%, tip swollen	***H. sobakini***
42	OLS & CS = 0.9 HD long, close at 0.6 HD apart; tail striated, conical-filiform, filiform part 66%, tip swollen	***H. ponticus***
–	OLS & CS = 0.8 HD long, at 0.8–1 HD apart; cuticle striated and with fine pores; tail conical- cylindrical, cylindrical part 20%, tip spherical swollen	***H. longinquus***
–	OLS & CS = 0.7–0.8 HD long, at 0.8 HD apart; cuticle smooth; tail conical-cylindrical, cylindrical part not striated 35–40%, tip club-shaped swollen	***H. minimus***
–	OLS & CS = 0.6 HD long, very close at 0.2 HD apart; cuticle smooth; head capsule present; tail conical-cylindrical, gradually tapering, tip swollen	***H. terrestris***
–	OLS & CS = 0.7 HD long, very close at 1.6 HD apart; cuticle thick and duplicated in head; very short amphid situated well behind, tail conical-cylindrical, cylindrical part 24%, tip blunt	***H. brachyaulax***
–	OLS & CS = 0.3 HD long, well separated at 1.3 HD apart; cuticle smooth; tail conical-cylindrical, cylindrical part 25%, tip round, swollen	***H. ciliocaudatus***
–	OLS & CS = 0.2–0.3 HD long, at 1 HD apart; cuticle smooth; tail conical-filiform, filiform part 50%, tip not swollen	***H. tenuicapitatus***

## Conclusions

Given the ecological significance of *Halalaimus*, from terrestrial environments to deep-sea regions potentially targeted by deep-sea mining such as the CCZ, the systematic framework presented here contributes not only to the taxonomy of the genus but also to broader research on (marine) biodiversity and conservation. In this study, we provide a comprehensive taxonomic review of the genus, including an overview of the morphological characteristics and the currently known geographical and bathymetrical distribution of all species. Notably, most exclusively deep-sea species were restricted to abyssal polymetallic nodule environments, although further research is needed to confirm this pattern. The updated species identification keys presented herein are essential tools for nematode taxonomists and species identifiers.

## Supplementary Material

XML Treatment for Halalaimus

XML Treatment for Halalaimus marcosi

XML Treatment for Halalaimus pierri

## Additional information

### Artificial Intelligence (AI) use

The authors accept full responsibility for the content of the manuscript, including the disclosure of any use of AI.

No AI tools were used in the preparation of this manuscript.

### Author ORCIDs

T.N. Bezerra https://orcid.org/0000-0002-4414-7827

N. Smol https://orcid.org/0000-0001-8635-726X

R.M.S. Alves https://orcid.org/0000-0002-8422-0611

E. Pape https://orcid.org/0000-0003-2881-8341

### Data availability

All of the data that support the findings of this study are available in the main text or Supplementary Information.

## Supplementary materials

Supplementary material 1 *Halalaimus* - Morphometrics Data type xlsx Explanation note Morphometrics of all described species of the genus *Halalaimus*. https://binary.pensoft.net/file/1705883This dataset is made available under the Open Database License (http://opendatacommons.org/licenses/odbl/1.0/). The Open Database License (ODbL) is a license agreement intended to allow users to freely share, modify, and use this Dataset while maintaining this same freedom for others, provided that the original source and author(s) are credited. Nic Smol, Ellen Pape, Tania Nara Bezerra

Supplementary material 2 *Halalaimus* - Distribution Data type xlsx Explanation note Distribution of all described species of the genus *Halalaimus*. https://binary.pensoft.net/file/1705884This dataset is made available under the Open Database License (http://opendatacommons.org/licenses/odbl/1.0/). The Open Database License (ODbL) is a license agreement intended to allow users to freely share, modify, and use this Dataset while maintaining this same freedom for others, provided that the original source and author(s) are credited. Renata Alves, Ellen Pape, Tania Nara Bezerra
